# Notch1 blockade by a novel, selective anti-Notch1 neutralizing antibody improves immunotherapy efficacy in melanoma by promoting an inflamed TME

**DOI:** 10.1186/s13046-024-03214-5

**Published:** 2024-11-04

**Authors:** Juliano Tiburcio de Freitas, Varsha Thakur, Kathryn M. LaPorte, Vijay S. Thakur, Brian Flores, Valentina Caicedo, Chioma G. E. Ajaegbu, Giuseppe Ingrasci, Zoe M. Lipman, Keman Zhang, Hong Qiu, Thomas R. Malek, Barbara Bedogni

**Affiliations:** 1 Department of Dermatology and Cutaneous Surgery, University of Miami Miller School of Medicine and Sylvester Comprehensive Cancer Center, 1600 NW 10th Ave, Miami, FL 33136 USA; 2 Department of Microbiology and Immunology, University of Miami Miller School of Medicine and Sylvester Comprehensive Cancer Center, Miami, FL 33136 USA; 3 Department of Radiation Oncology, University of Miami Miller School of Medicine and Sylvester Comprehensive Cancer Center, Miami, FL 33136 USA; 4 Department of Biochemistry, Case Western Reserve University, Cleveland, OH 44106 USA

## Abstract

**Background:**

Immune checkpoint inhibitors (ICI) have dramatically improved the life expectancy of patients with metastatic melanoma. However, about half of the patient population still present resistance to these treatments. We have previously shown Notch1 contributes to a non-inflamed TME in melanoma that reduces the response to ICI. Here, we addressed the therapeutic effects of a novel anti-Notch1 neutralizing antibody we produced, alone and in combination with immune checkpoint inhibition in melanoma models.

**Methods:**

Anti-Notch1 was designed to interfere with ligand binding. Mice were immunized with a peptide encompassing EGF-like repeats 11–15 of human Notch1, the minimal required region that allows ligand binding and Notch1 activation. Positive clones were expanded and tested for neutralizing capabilities. Anti-Notch1-NIC was used to determine whether anti-Notch1 was able to reduce Notch1 cleavage; while anti-SNAP23 and BCAT2 were used as downstream Notch1 and Notch2 targets, respectively. K457 human melanoma cells and the YUMM2.1 and 1.7 syngeneic mouse melanoma cells were used. Cell death after anti-Notch1 treatment was determined by trypan blue staining and compared to the effects of the gamma-secretase inhibitor DBZ. 10 mg/kg anti-Notch1 was used for in vivo tumor growth of YUMM2.1 and 1.7 cells. Tumors were measured and processed for flow cytometry using antibodies against major immune cell populations.

**Results:**

Anti-Notch1 selectively inhibited Notch1 but not Notch2; caused significant melanoma cell death in vitro but did not affect normal melanocytes. In vivo, it delayed tumor growth without evident signs of gastro-intestinal toxicities; and importantly promoted an inflamed TME by increasing the cytotoxic CD8^+^ T cells while reducing the tolerogenic Tregs and MDSCs, resulting in enhanced efficacy of anti-PD-1.

**Conclusions:**

Anti-Notch1 safely exerts anti-melanoma effects and improves immune checkpoint inhibitor efficacy. Thus, anti-Notch1 could represent a novel addition to the immunotherapy repertoire for melanoma.

**Supplementary Information:**

The online version contains supplementary material available at 10.1186/s13046-024-03214-5.

## Introduction

Immune checkpoint inhibitor (ICI) therapy has revolutionized the treatment of metastatic melanoma patients. Recent data from the CheckMate-067 phase III trial reported overall survivals (OS) of 57% in the combination ipilimumab/nivolumab, 43% in nivolumab monotherapy, and 25% ipilimumab monotherapy at 6.5 years [1]. However, resistance to ICI therapies is relatively common, with 55% of melanoma patients presenting innate resistance to anti-PD-1; 40% to anti-PD-1/anti-CTLA-4 combination; and 25% patients developing resistance to anti-PD-1 within two years of treatment [2].

Response to ICIs can be dictated by both tumor intrinsic factors as well as those associated with the tumor microenvironment (TME). Several tumor intrinsic oncogenic pathways have been shown to mediate T cell exclusion. For example, melanomas with increased WNT/β-catenin activation lacked tumor-infiltrating T cells, mimicking the *non-inflamed* phenotype leading to resistance to ICI and adoptive T cell transfer [3, 4]. Also, loss of the tumor suppressor *PTEN* in metastatic melanoma correlates with decreased intratumoral T cell infiltration and reduced responsiveness to PD-1 inhibitor therapy [5]. In mouse models, PI3K inhibition improved the efficacy of ICI [5].

Regarding the TME, responses to immunotherapy preferentially occur in tumors with a preexisting antitumor T cell response. Typically, these tumors present high CD8^+^ T cells and contain proinflammatory cytokines that provide a more favorable environment for T cell activation and expansion, i.e. an IFNγ signature, high IL-12, IL-23, IL-1β, TNF-α, IL-2. Such tumors are define as *T cell inflamed* as opposed to *non-inflamed* tumors which lack expression of IFNγ and CD8^+^ T cell signatures and generally express cytokines associated with immune suppression or tolerance (e.g. IL-10, TGFβ); and can contain high levels of immunosuppressive cells (i.e. regulatory T cells – Tregs, and myeloid-derived suppressor cells - MDSCs) [6]. Finally, tumor associated B cells promote intra-melanoma inflammation that favors response to ICIs [7]. Therefore, a baseline inflamed tumor microenvironment correlates with responsiveness to checkpoint blockade, while the non-inflamed phenotype correlates with treatment resistance [8].

We have previously shown that Notch1-expressing melanomas are characterized by an immune-suppressed, non-inflamed tumor microenvironment enriched in Tregs, MDSCs and immunosuppressive cytokines [9].

Notch1 is an evolutionarily conserved signaling cascade with critical roles in the maintenance of melanocyte stem and precursor cell homeostasis [10]. However, Notch1 signaling is reactivated in several cancers including melanoma. We and others have shown that over 60% of melanomas, irrespective of driver mutation status (BRAF, RAS) express active Notch1, and that Notch1 is associated with poorer outcome and progression. Finally, Notch1 has been shown to be required for tumor intrinsic functions such as growth and metastasis [11–16].

Our previous work demonstrated that RNAi-mediated Notch1 inhibition promoted an inflamed TME by reducing immunosuppressive cytokines and factors (e.g., IL10, TGFβ, ARG1), immunosuppressive cells (MDSCs and Tregs); and by increasing IFNγ and CD8^+^ T cells in the melanoma TME. These changes resulted in a reduced Tregs/CD8 ratio and better responses to ICIs [9].

Pan-Notch inhibitors such as γ-secretase inhibitors (GSIs), have been tested in several preclinical models of cancer, including melanoma [11, 16, 17]; as well as employed in clinical trials [17]. However, despite anti-tumor activity in the preclinical setting, no significantly improved outcomes have been observed in patients treated with GSI alone or in combination with standard of care [17]. One caveat might have been the lack of immunogenic models to study the role of GSIs on the immune TME. For example, maintenance of Notch2 function is needed to ensure the cytotoxic activity of CD8^+^ T cells. Loss of Notch2 in CD8^+^ T cells (but not of Notch1), has been shown to promote tumor growth in mice [18]; and blockade of nuclear translocation of Notch2 intracellular domain (NIC), the active form of Notch, inhibits the cytotoxic efficacy of CD8^+^ T cells on hepatocellular carcinoma [19]. Finally, the toxicity of GSIs can also be a limitation. Blockade of both Notch1 and 2, which are required for the maintenance of stem cells in the intestinal crypts [20–22], causes GI toxicities requiring careful assessment of therapeutic regimens and careful monitoring of patients; and certain GSIs may promote non-melanoma skin cancer [23]. Thus, selective inhibition of Notch1 may be preferable to pan-Notch inhibition.

Here we show that inhibition of Notch1 by a novel, selective neutralizing monoclonal antibody (anti-N1) developed in our laboratory, delays melanoma growth; promotes an inflamed TME which enhances the efficacy of anti-PD1; and is devoid of the side effects observed with GSI. Anti-N1 could represent a novel addition to the immunotherapy repertoire for melanoma.

## Materials and methods

### Cell lines, tissue specimens and reagents

Melanoma cells used in this study were: human K457, A375, SKMel2, MeWo and WM-266-4 (a gift from Dr Marianne Broome Powell, Stanford University) [16, 24]; mouse YUMM2.1 (BRAF^V600E^/WT; PTEN-/-; CDKN2+/-; BCAT^loxex3^/WT) and YUMM1.7 (BRAF^V600E^/WT; PTEN-/-; CDKN2-/-) (a gift from Dr. Marcus Bosenberg) [25, 26]. Cells were maintained in DMEM (Dulbecco’s modified Eagle’s medium) supplemented with 10% fetal calf serum, 1% glutamine and 1% penicillin–streptomycin (Pen/Strep).

Chart review to identify melanoma samples from immunotherapy responders and non-responders was done on de-identified melanoma tissues obtained from the Sylvester Comprehensive Cancer Center Biobank at the University of Miami (IRB# MOD00041812).

DBZ (dibenzazepine) was purchased from Selleck Chemicals (Houston, TX, USA). *InVivo*Plus anti-mouse PD-1 (CD279) and *inVivo*Plus rat IgG2a isotype control were purchased from BioXCell (Lebanon, NH) and used at 100 µg per dose per mouse.

### Antibody generation

A GST-tagged peptide encompassing EGF-like repeats 11–15 of human Notch1 was generated using pGEX in E.coli. Prior to immunization, GST was removed by thrombin digestion. Female Balb/c mice (8–10 weeks old) were used for immunization. A 1:1 volume of Freud’s Adjuvant (Sigma) with antigen was injected I.P. on day 0, 10 and 20. Ag8.653 myeloma cells, maintained in complete RPMI, were used for fusion. Splenocytes and myeloma cells were fused using PEG in HAT media (RPMI with 20% FBS, HAT or HT(1: 500); Pen/Strep and Gentamicin). Six positive clones were identified by indirect ELISA. Clone 1B6 was chosen and expanded. Antibody was produced by the ascites method and purified using Melon Gel (Thermo Fisher Scientific) as per manufacturer’s instructions.

### Western blot analysis

Cells (1 × 10^6^) were plated in complete media containing vehicle (DMSO) and/or IgG, DBZ (10 µM) and anti-Notch1 (25 µg/ml), and collected 48–72 h after treatment. Total protein was extracted with urea lysis buffer (9 M urea, 75 µM Tris-HCl, pH 7.5, and 100 µM 2-mercaptoethanol). 40–50 µg protein per sample was separated by 8–10% SDS-PAGE and transferred onto PVDF membranes. Anti cleaved-Notch1 (Val1744) was from Cell Signaling Technology (Beverly, MA); anti SNAP23 andBCAT2 were from Proteintech (Rosemont, IL 60018, USA). Loading was normalized with anti–b-actin, GAPDH, or a-tubulin (Santa Cruz Biotechnology, Dallas, TX).

### Cell survival

Cell survival/death was calculated by counting cells (T20 cell counter, Biorad) using trypan blue, after three days incubation with the indicated reagents. Colony assays were performed by seeding 1000 cells per well (6-well plate). Cells were left to adhere for 24 h, then reagents were added. After 10 days incubation, colonies were stained with crystal violet and scored using GelCount (Oxford Optronix).

### Melanoma spheroids 

K457 cells (200 µl, 25,000/ml) were added to a 96-well plate coated with 1.5% agar (Difco, Sparks, MD). Cells were incubated for 3 days to allow formation of spheroids. Spheroids were harvested and implanted into collagen I/complete media gels containing the indicated reagents. After 3 days of incubation, spheroids were washed in phosphate buffer saline and stained with calcein-AM and propidium iodide (Molecular Probes, Eugene, OR) for 1 h according to the manufacturer’s instructions. Pictures were taken using a Keyence inverted fluorescence microscope.

### In vivo tumor growth

YUMM2.1 and YUMM1.7 cells (5 × 10^5^) were inoculated subcutaneously in the dorsal flanks of 8-week-old C57BL/6 male mice (Charles River) for a total of ten tumors per experimental group. Mice were treated with IgG (10 mg/Kg), anti-N1 (10 mg/Kg), anti-PD1 (100 µg/mouse [27] or DBZ (10umol/mouse), delivered every other day or delivered three consecutive days followed by four days holiday period, for DBZ [16]. Tumors were measured at least twice weekly with a caliper and tumor volumes calculated as (*w*^2^ × *l*) × 0.52, in which *w* and *l* represent width and length, respectively [11].

### Flow cytometry

Tumors were harvested at the end time point and were dissociated using collagenase (Sigma), DNase (Sigma) and trypsin (Gibco). Red blood cells were removed from cell suspension with ACK Lysis buffer (Thermo Fisher Scientific). Cells were counted and an equal number of cells were incubated prior to flow cytometry with the following antibodies: CD45, CD3, CD69, CD25, CD11b, Ly6C, Ly6G (Biolegend) and CD8, CD4, FOXP3 (BD Biosciences). Live/Dead Blue dye (Thermo Fisher Scientific) was employed to exclude dead cells from flow cytometry analysis. Data was acquired using Aurora (Cytek) flow cytometer and analyzed using the FlowJo (TreeStar) software.

### Preparation of tumor-infiltrating lymphocytes (TILs)

YUMM2.1 melanoma tumors (*n* = 5) were treated for 14 days with either IgG or anti-N1 (10 mg/Kg, every other day). Tumors were then excised, weighed, minced into 1–2 mm pieces, and incubated at 37 °C with gentle shaking in RPMI-CM containing 1 mg/mL Collagenase (Sigma cat. #C5138) for 20 min. Single cell suspensions were obtained using gentle-MACS C Tubes (Miltenyi Biotec, cat. #130096334) and the gentle MACS Dissociator. Single cell suspensions were washed 1× with RPMI- CM to remove Collagenase. Cell pellets were resuspended with 2 ml ACK lysis buffer for 3 min in a 37 °C water bath, then washed 1× with HBSS. Cell pellets were resuspended in 10 mL RPMI-CM, passed through a 70 μm filter, counted, and prepared for CD45 enrichment. Mouse CD45 microbeads (Miltenyi Biotec, cat. #130-052-301), LS columns (Miltenyi Biotec, cat. #130-042-401), and a quadroMACS Separator (Miltenyi Biotec, cat. #130-091-051) were used per manufacturer’s recommendations to isolate CD45-expressing immune cells from the TME for functional assays.

### Generation of bone marrow-derived dendritic cells

Femur bones were collected from C57/Bl6 mice, muscle tissue removed, and bones sterilized in a petri dish containing 70% ethanol. The bones were then washed with PBS and then crushed in cold 1× HBSS via a pestle and collected by centrifugation (1200 rpm, 5 min, 4 °C). Cell pellets were resuspended in 2 mL ACK lysing buffer, incubated for 2 min, and washed with HBSS. The cell pellets were resuspended in 5 mL of RPMI-CM and filtered through a 70 μm cell strainer. Bone marrow cells (10 × 10^6^) were cultured in 10 mL RPMI-CM containing 20 ng/mL recombinant mouse GM-CSF (rmGM-CSF, PeproTech, Cat. No. 315–03) using 100 × 15 mm culture dishes (VWR, Cat. No. 25 384–342) at 37 °C in a humidified atmosphere at 5% CO 2. On day 3, an additional 10 mL of RPM1-CM containing 20 ng/mL rmGM-CSF was added to each plate. On days 6 and 8, 50% of the media was removed from each plate and any non-adherent cells were collected by centrifugation, pellets resuspended in 10 ml RPMI-CM containing 20 ng/mL rmGM- CSF and added back to the corresponding culture dish. To mature the DCs, on day 10, non-adherent cells from each culture plate were again harvested, centrifuged, and pellets resuspended in 10 ml RPMI-CM containing 10 ng/ml rmGM- CSF and 1 µg/ml LPS (lipopolysaccharides from Escherichia coli O55:B5, Millipore Sigma, cat. No. L2880) and re-plated in a new 100 × 15 mm culture dish. On day 11, 10 ml RPMI- CM was added to each dish. On day 12, adherent bone marrow- derived DCs (BMDCs) were collected using a cell scraper, frozen in 90% FBS and 10% dimethyl sulfoxide (DMSO) and stored at − 150 °C until used in vitro.

### Ex vivo assay

TILs were isolated from control and treated tumors, were stimulated with PMA and ionomycin and co-cultured with APCs (10:1 effector to APC) and naïve melanoma cells (5:1 effector to melanoma cells) in the presence or absence of anti-N1 for 12 h. Trypan blue staining was employed to determine the cytotoxicity of isolated TILs. IFNγ and GrzB expression in co-cultures were assessed using Mouse ELISpot Development Module (R&D Systems). CD8^+^ T cells were analyzed through flow cytometry to evaluate the expression of effector and degranulation molecules via intracellular cytokine staining of IL-2, TNFα, IFNγ, and GrzB (Biolegend) and extracellular CD107a (Biolegend) staining.

### CD107 degranulation assay

Cells were incubated for 4 h with GolgiStop (BD Biosciences) to inhibit protein transport. Next, cells were stained with CD45, CD8, CD3, CD107a, and CD11c (Biolegend) surface markers. Following surface staining, cells were fixed and permeabilized using Cytofix/Cytoperm (BD Biosciences). Then, intracellular staining of IL-2, TNFα, IFNγ, and GrzB (Biolegend) cytokines was performed in presence of permeabilization buffer (BD Biosciences). Quantification of all markers and cytokines was performed by flow cytometry.

### Enzyme-linked immunospot assay

ELISpot assays were performed following manufacturer’s protocols using the Mouse IFNγ and GranzymeB Enzyme-Linked ImmunoSpot (ELISpot) sets (BD Biosciences). In brief, serial dilutions of lymphoid cells from cultures or TILs were cultured in RPMI-CM with or without AH1 tumor antigen peptide (2 µg/mL; ChiScientific, sequence: SPSYVYHQF) and 10-fold lower numbers of BMDCs in anti-IFNγ or GrzB coated 96-well ELISpot plates (Millipore Sigma) for 19–24 h at 37 °C in 5% CO2. Secretion of IFNγ and GrzB was detected using biotinylated anti-IFN-γ or anti-GrzB (BD Biosciences) followed by the addition of Streptavidin-HRP (1:100, BD Biosciences) and AEC Chromogen/Substrate (BD Biosciences). ELISpots were visualized using Immuno-Spot S6 Universal and quantified using Immuno-Spot V.7.0.30.4 Analyzer Professional DC software.

### Immunohistochemistry

Immunohistochemistry was performed on formalin-fixed paraffin-embedded (FFPE) tissue after Tris-based buffer (pH 9.0) retrieval using Antigen Unmasking Solution (Vector Labs). Sections were incubated with blocking buffer for 1 h at room temperature (Vector Labs), followed by primary antibody incubation overnight at 4 °C, then incubated for 30 min with ImmPRESS (Peroxidase) Polymer Reagent (Vector Labs). ImmPACT DAB (Vector Labs) was used as chromogen and slides were imaged and recorded using a Keyence microscope.

HES1 IHC staining was quantified using the Fiji (ImageJ) software as described by Crowe and Yue [28]. Briefly, deconvolution was applied to the IHC images to separate DAB (3,3’-diaminobenzidine) signal from other staining components. Next, the DAB intensity threshold was set up to distinguish between positive DAB staining and background. Positive control images were used to ensure specificity. Then, DAB signal was quantified as mean grey value within defined regions of interest.

### Statistical methods

Data were statistically analyzed using the Student t test for comparisons between two groups and the Multiple Student’s t test when comparing multiple groups in GraphPad Prism 10.0. The results were expressed as mean ± standard error. A P value < 0.05 was considered statistically significant.

## Results

### Notch1 pathway correlates with a non-inflamed gene signature in melanoma and is preferentially elevated in non-responder patients

**Fig. 1:**
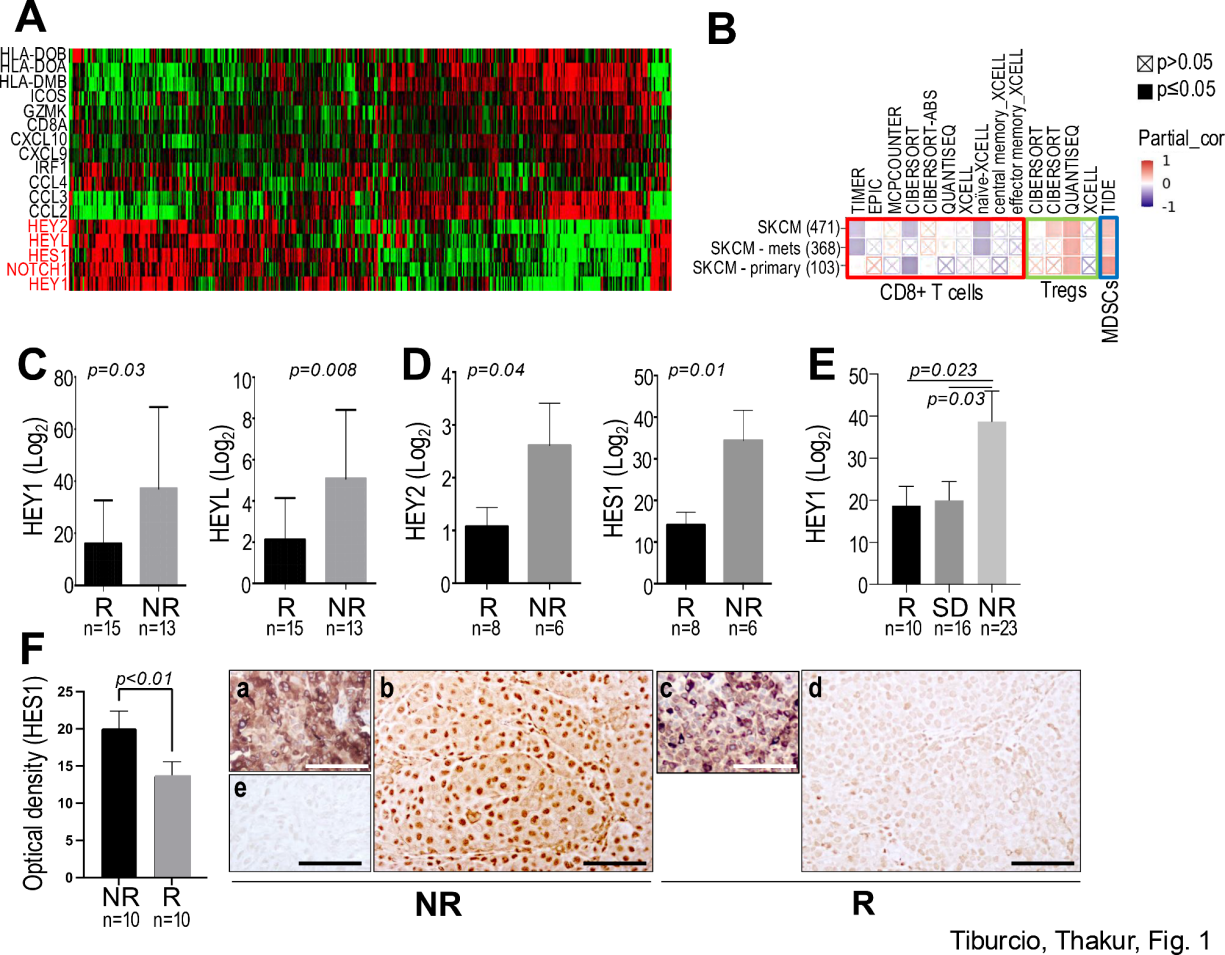
Notch1 signaling inversely associates with the inflamed status of melanomas and response to anti-PD1 therapy. (**A**) T-cell inflamed gene signature derived from *Spranger et al.* (3), compared to Notch1 and Notch target genes HEY1, HEY2, HEYL. The expression of Notch1 and the Notch1 downstream genes inversely correlates with inflamed and non-inflamed tumors (TCGA melanoma data set https://tcga.xenahubs.net/download/TCGA.SKCM.sampleMap/HiSeqV2.gz). Notch pathway genes are highlighted in red. *n* = 474. **(B)** Timer2.0 immune infiltration in TCGA melanoma tumors in correlation with Notch1 gene expression. Spearman’s correlation, *p* < 0.05. Significant correlation is in bold color, non-significant is crossed. **(C)** HEY1 and HEYL expression is higher in patients that progressed on anti-PD-1 therapy in the GEO data set GSE78220. **(D)** HEY2 and HES1 expression is higher in patients that did not respond to anti-PD-1 therapy in the GEO data set GSE78220. **(E)** HEY1 expression is higher in patients that progressed on anti-PD-1 therapy (GEO data set GSE91061). **(F)** FFPE sections from the Sylvester biobank corresponding to anti-PD-1 and or anti-CTLA-4 or combination treated patients, that either responded or progressed. Immuno-staining: (**a, c**): anti-melanA; (**b, d**): anti-HES1; (**c**) negative control. Scale bar: 100 μm. Responders (R): partial and complete response; SD: stable disease; Non-responders (NR): progressive disease

We have previously shown that Notch1 expressing melanomas are characterized by an immune-suppressed, non-inflamed tumor microenvironment enriched in Tregs, MDSCs and immunosuppressive cytokines [9]. To support these experimental data of a role of Notch1 in promoting a non-inflamed TME, we interrogated in silico data from the melanoma TCGA (*n* = 474), and compared the T cell inflamed gene signature from *Spranger et al.* [3], with a Notch signature. Figure 1A shows that higher expression of Notch1 and of the Notch target genes HEY1, HEY2 and HEYL and HES1 associates preferentially with non-inflamed melanomas, whereas lower Notch1 and Notch target genes associates with inflamed melanomas. To further support this observation, the potential correlation between Notch1 and immune infiltrates was investigated using TIMER 2.0 (Tumor IMmune Estimation Resource), which integrates six state-of-the-art algorithms, including TIMER, xCell, MCP-counter, CIBERSORT, EPIC and quanTIseq for deconvolution (reviewed in [29]). The heat map, showing only melanoma samples (total, metastatic and primary), shows that Notch1 inversely correlates with CD8^+^ T cells, while it positively correlates with Tregs and MDSCs (Fig. 1B). A complete heat map depicting all TCGA tumors and representative correlation analyses of Notch1 and immune infiltrates in melanoma is shown in Suppl. Figure 1.

These data further support the notion that Notch1 signaling upregulation promotes a non-inflamed TME, which is likely to be less responsive to ICIs. To address the correlation between ICI response and Notch signaling activation, publicly available RNAseq data sets of melanoma patients that either did or did not respond to ICIs were interrogated. We found that the expression of the Notch1 downstream targets HEY1, HEY2, HEYL and HES1, are significantly higher in non-responders (NR) than in responders (R) among patients that underwent anti-PD-1 treatment [30–32] (Fig. 1C-E). To further support the in-silico data, tumor sections obtained from melanoma patients that underwent anti-PD-1 and anti-CTLA-4 treatment at UM Medical Campus-Sylvester Comprehensive Cancer Center, were immuno-stained with anti-HES1 (Fig. 1Fb, d). Notably, HES1 staining intensity, evaluated by quantifying the optical density of the nuclear staining, was significantly higher in non-responders than in responders. MelanA positivity confirm the samples are melanoma (Fig. 1Fa, c).

Overall, these data support a critical role of Notch1 signaling in melanoma response to ICI and may potentially represent a predictor of ICI response in melanoma patients.

### A novel anti-Notch1 monoclonal antibody inhibits Notch1 activation without affecting Notch2

Pan-Notch inhibitors, such as γ-secretase inhibitors (GSIs), have been extensively studied in preclinical models of cancer driven by elevated Notch signaling [17]. However, apart from brain tumors, they showed no significant benefit in clinical trials for several cancer types [17]. Additionally, GSIs are non-selective, inhibiting all Notch receptors. Particularly, inhibition of both Notch1 and Notch2 is responsible of GI toxicities observed in both animal models and patients, while Notch2 is required by CD8^+^ T cells for their anti-tumor cytotoxicity [18, 19]. Finally, our previous work supported the specific role of Notch1 in promoting a tolerogenic TME [9].

**Fig. 2:**
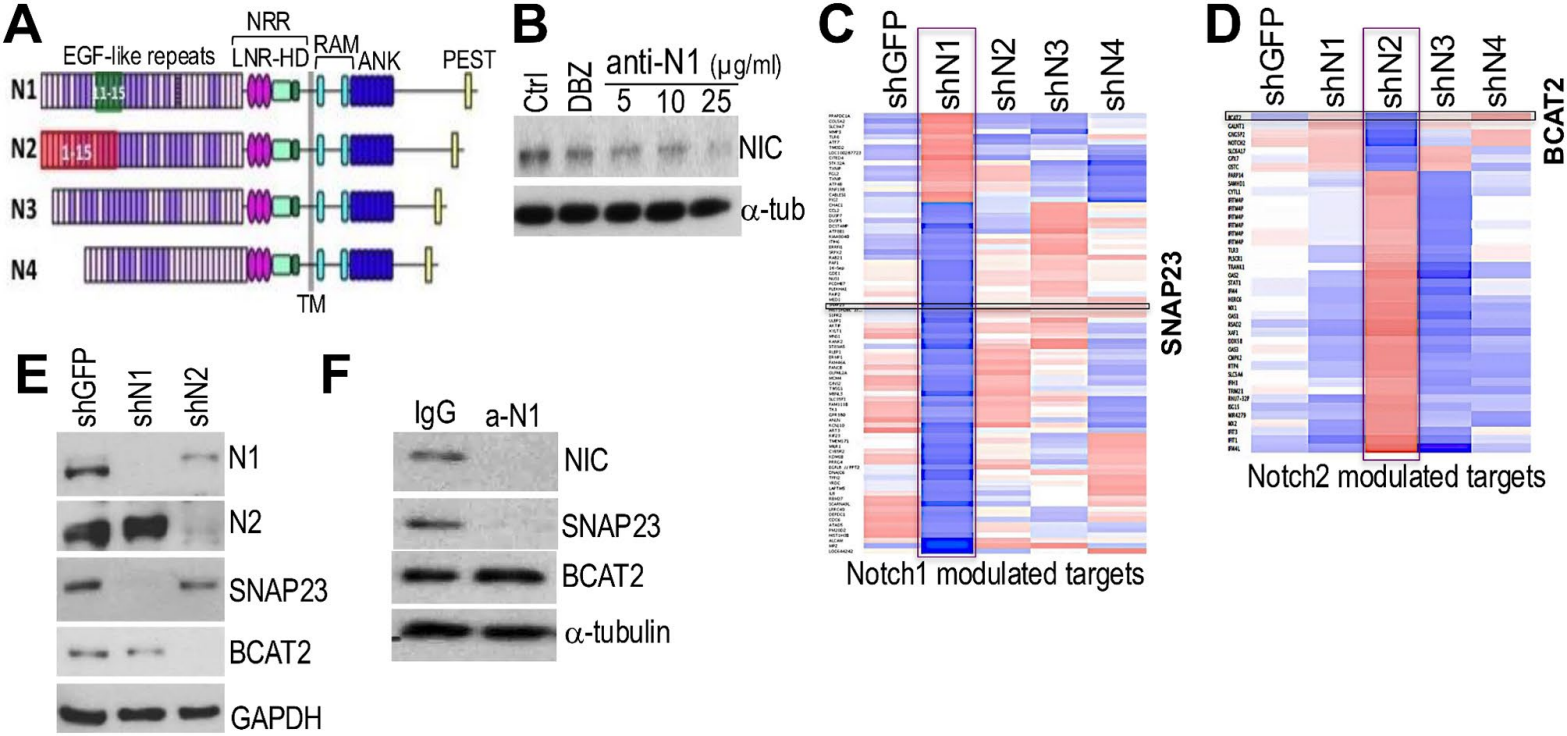
Anti-N1 selectively inhibits Notch1. **(A)** EGF-like repeats 11–15 and 1–15 of Notch1 and 2. **(B)** K457 melanoma cells treated for 72 h with DBZ (5 μm) and 3 doses of anti-N1. **C**,** D)** Microarray analysis of WM266-4 cells expressing shGFP or shRNAs against each Notch receptor. Notch1 (**C**) and Notch2 (**D**) only modulated genes. SNAP23 and BCAT2 are shown. **E)** Total Notch1, Notch2, SNAP23 and BCAT2 expression in K457 cells expressing shNotch1 or shNotch2. **F)** Cleaved Notch1, SNAP23 and BCAT2 in K457 cells treated with IgG control or anti-N1 (25ug/ml) for 72 h

Therefore, to block Notch1 selectively without affecting other Notch receptors, predominantly Notch2, we designed an anti-Notch1 (anti-N1) neutralizing antibody that interferes with ligand binding. The binding of the ligand is required to expose the S2 site to protease (TACE) cleavage, a step that must occur in order to allow cleavage by γ-secretase, which releases the intracellular domain, the active form of Notch1 [33]. The antibody was produced using a peptide spanning EGF-like repeats 11–15 (Fig. 2A) to include the minimal required region that allows ligand binding and Notch1 activation [34]. Alignment of this region with the minimal EGF-like repeats ligand binding region of Notch2 (EGF-like repeats 1–15 [34]) revealed 64% similarity (blast.ncbi.nlm.nih.gov), indicating reduced probability of cross reactivity with Notch2. Notch1 activation after treatment with anti-N1 was tested with anti-cleaved Notch1-Val1744 (Cell Signaling), which recognizes the γ-secretase cleaved NIC. anti-N1 reduced active (cleaved) Notch1 in a dose-dependent manner (Fig. 2B). The GSI dibenzazepine (DBZ) was used as positive control and showed reduced Notch1-NIC, as expected. To assess Notch1 and Notch2 activity, we used SNAP23 and BCAT2, two downstream targets of Notch1 and 2, respectively, that we identified by a microarray analysis of human melanoma cells in which each Notch receptor was inhibited by a specific shRNA (Fig. 2C, D). We have previously identified SNAP23 (Synaptosome Associated Protein 23) as a regulator of CCL5 secretion downstream of Notch1 [9]. BCAT2 (Branched Chain Amino Acid Transaminase 2) was identified as a Notch/RBPJ predicted target (http://amp.pharm.mssm.edu/Harmonizome/resource/ TRANSFAC) [35, 36]. SNAP23 and BCAT2 are exclusively controlled by Notch1 and 2, respectively, as shown by RNAi inhibition of either receptor (Fig. 2E). Importantly, anti-N1 inhibited only SNAP23 but not BCAT2, indicating antibody selectivity (Fig. 2F).

To further support the selective targeting of Notch1, we compared our monoclonal antibody (MAb) to brontictuzumab (BRON), a *first-in-human* tested blocking Mab against Notch1. Surprisingly, however, BRON not only failed to block Notch1 in melanoma cells, but also failed to affect cell survival (Suppl. Figure 2). BRON was designed to recognize the Negative Regulatory Region (NRR) of Notch1, which is the region where gain-of-function mutations have been reported in 50% of T cell acute lymphoblastic leukemia (T-ALL). These mutations disengage the heterodimerization domain (HD) leading to ligand-independent receptor activation [37]. BRON was indeed effective against T-ALL cells derived from patients [38], however, had limited anti-tumor efficacy in a phase 1 clinical trial for patients with different cancer types, with only 6 out of 36 patients assessed showing partial response and stable disease [39]. It is possible that in a cell system relying on ligand binding for Notch1 activation, a neutralizing antibody blocking the ligand binding domain (LBD) may be more effective. Indeed, studies comparing LBD and NRR Notch1 antibodies showed the NRRs were incomplete antagonists [40].

### Anti-N1 exert cytotoxic activity

**Fig. 3:**
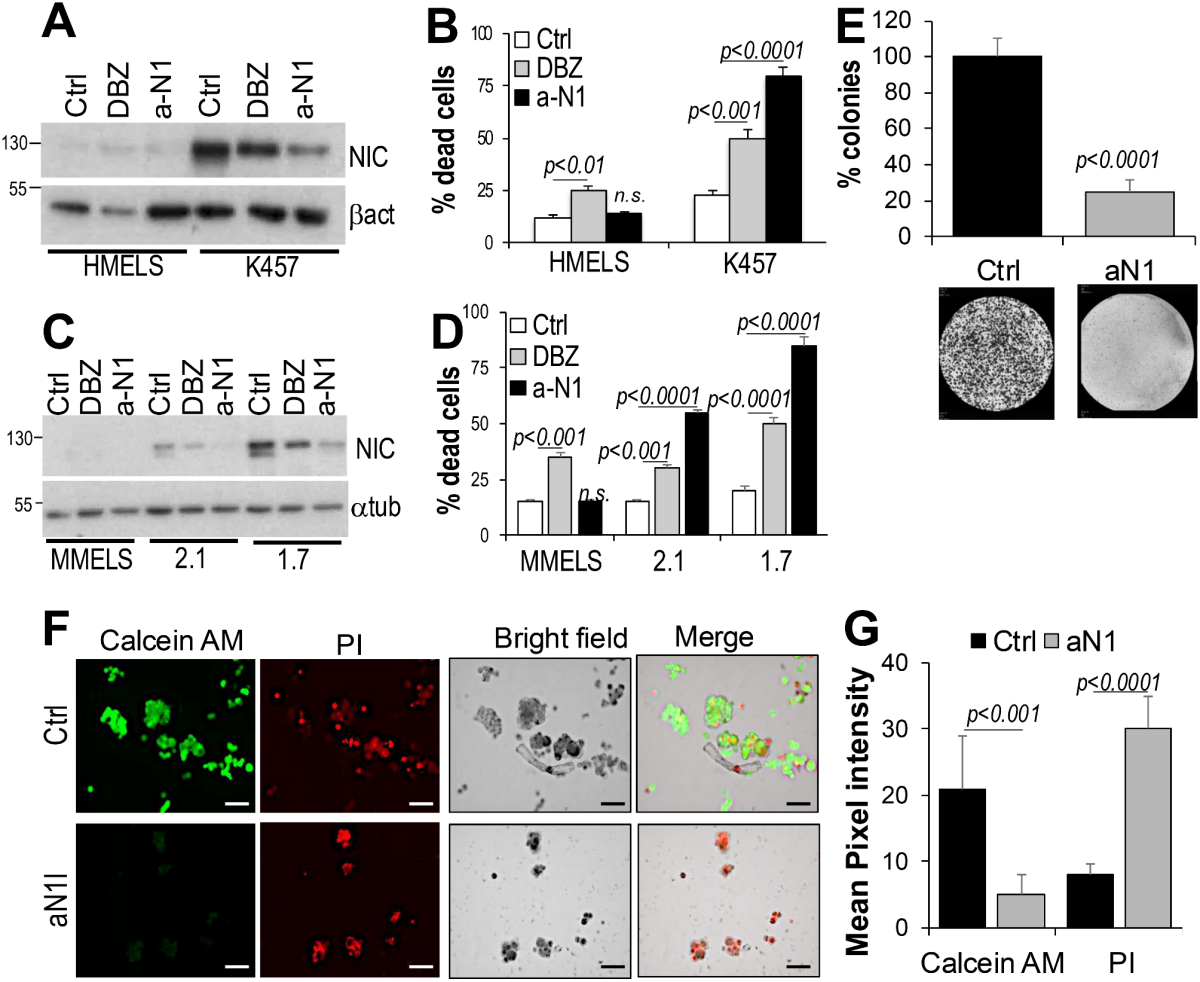
Anti-N1 causes melanoma cell death. **(A)** Notch1 expression in human (HMELS) melanocytes versus K457 melanoma cells, treated with IgG/DMSO, DBZ (10 μm) or anti-N1 (25ug/ml) for three days. **(B)** % cell death of the cells in A, measured by trypan blue. **(C)** Notch1 expression in mouse (MMELS) melanocytes versus YUMM2.1 and YUMM1.7 mouse melanoma cells, treated with IgG/DMSO, DBZ (10 μm) or anti-N1 (25ug/ml) for three days. **(D)** % cell death of the cells in C, measured by trypan blue. **(E)** Clonogenic assay of K457 cells treated with IgG or anti-Notch1 (25ug/ml). Colonies were counted after 10 days from seeding. **F**,** G)** 3D Spheroids were grown in collagen type I, then treated with IgG or anti-N1 (25ug/ml) for three days. Calcein AM and propidium iodide were added and green (alive) and red (dead) cells were assessed by quantifying the pixel intensity of each well. A minimum of 100 spheroids were counted. Data are the mean of three independent experiments each performed in triplicate. Scale bars: 100 μm

We and others have previously shown that Notch1 inhibition delays tumor growth and promotes cell death [11, 13, 14, 16]. Therefore, we tested the cytotoxic abilities of the antibody in culture. A three-day treatment with anti-N1 induced 75%, 80% and 55% cell death in the K457 (human) and the YUMM1.7 and YUMM2.1 (mouse) melanoma lines, respectively (Fig. 3A-D), but did not affect either human or mouse melanocytes, which express low or undetectable Notch1 levels compared to melanoma cells. This indicates potential Notch1 addiction of melanoma. Additional human melanoma cell lines (A375, SKMel2 and MeWo), demonstrated 80%, 40% and 60% cell death respectively, upon anti-N1 treatment (Suppl. Figure 3). Though the response varies among cell lines, possibly due to the level of Notch1 expression and therefore dependency on the signaling pathway, these data indicate inhibiting Notch1 promotes melanoma cell death. DBZ showed a significant killing effect on both melanoma cells and slightly on melanocytes, suggesting potential toxicity to normal cells. Indeed, treatment of normal human fibroblast and HaCaT with DBZ and anti-N1, revealed that while DBZ was cytotoxic, especially in fibroblasts, anti-N1 did not affect survival of either cell type (Suppl. Figure 4).

To further support the cytotoxic activity of the antibody against melanoma, we performed a clonogenic assay and found that the antibody reduced the number of viable colonies by 80% (Fig. 3E). Finally, to mimic the 3D structure of the tumor, K457 melanoma cells were grown into spheroids as previously shown [16], and then treated with anti-N1. After three days in culture, Calcein AM and Propidium iodide were added to detect alive and dead cells, respectively. Pixel intensity was used as indirect quantification of alive (green) and dead (red) cells and showed a 4-fold reduction in alive cells, and a correspondent 4-fold increase in dead cells (Fig. 3F, G) in the anti-N1 treated groups.

Overall, these data indicate anti-N1 is cytotoxic against melanoma cells, but safe for normal cells, supporting the selectivity and anti-cancer activity of anti-N1.

### Anti-N1 exert anti-tumor activity in vivo

**Fig. 4:**
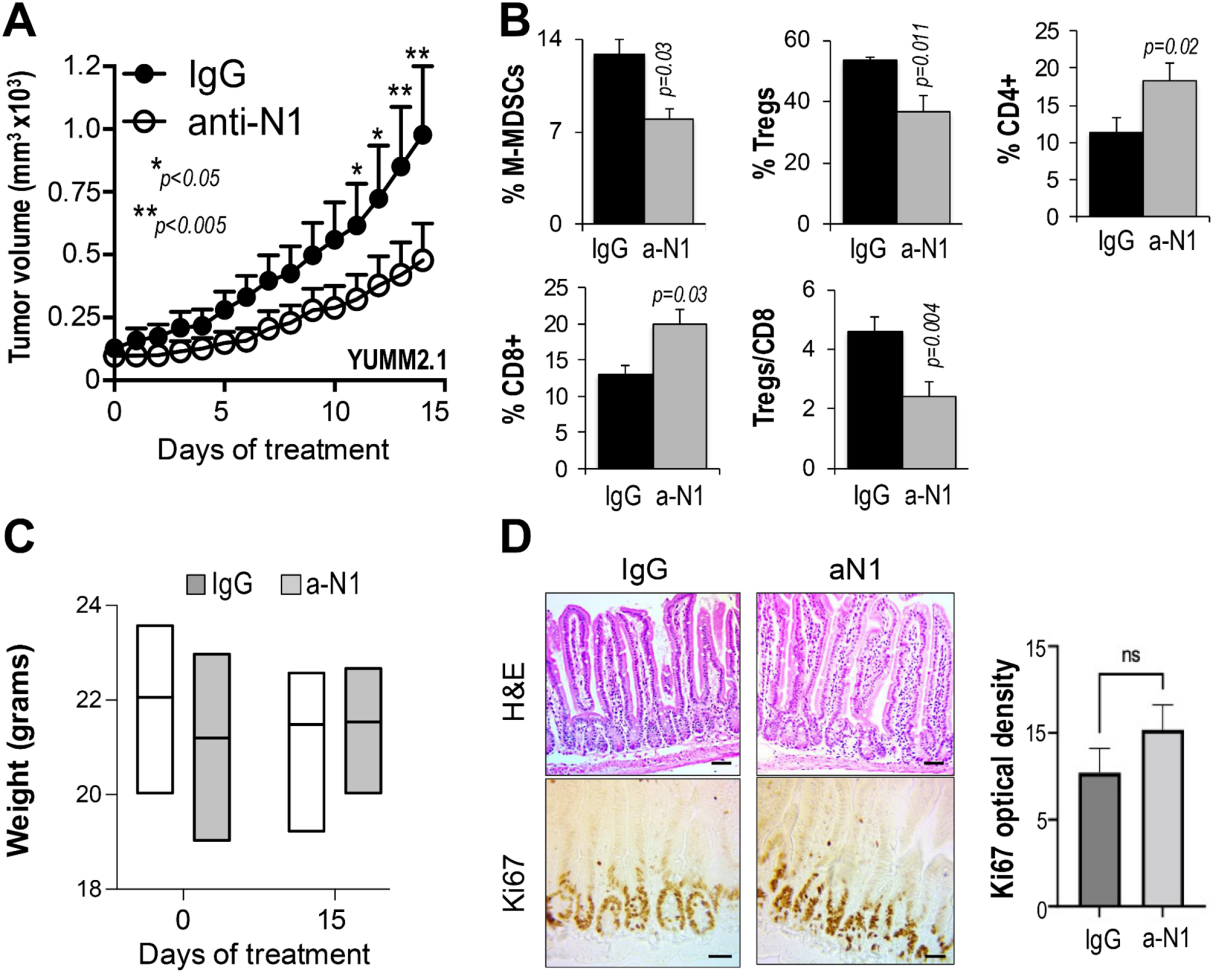
Anti-N1 delays tumor growth and promotes an inflamed TME: **(A)** Growth rates of YUMM2.1 tumors treated with IgG or anti-N1 (10 mg/Kg) every other day. Data are the mean ± SEM, of two experiments performed independently, each containing 10 tumors per group. **(B)** % M-MDSCs (CD11b^+^; Ly6C^hi^; ly6G^−^ = monocytic), Tregs (CD4^+^/FoxP3^+^), CD4^+^ T cells, CD8^+^ T cells, in the TME of YUMM2.1 tumors from the mice treated in A. The Tregs/CD8 ratio was calculated by dividing the absolute number of CD4^+^/FoxP3^+^ and CD8^+^ T cells in tumors. Absolute numbers were obtained by normalizing the number of cells detected by flow cytometry to the tumor mass. **(C)** Mouse weight at time 0 and at the end time point of 15 days. No significant differences were observed among groups (IgG vs. aN1) and between groups at time 0 and end time point (*p* > 0.05). **(D)** H&E and Ki67 staining of sections of intestines from the mice in A, collected at the end time point. Left: representative pictures; right: quantification of pixel intensity of Ki67 staining. *n* = 10. Scale bar: 100 μm

To thoroughly test the anti-tumor activity of anti-N1, the mouse syngeneic melanoma lines YUMM2.1, which respond to ICI, and YUMM1.7, which is instead resistant [25, 26], were inoculated subcutaneously in C57Bl/6 mice, which retain an intact immune system, allowing analysis of Notch1 blockade on the TME. Once tumors in all mice reached approximately 100-150mm^3^ in volume, measured with a caliper, the animals were divided into 2 groups: control (IgG, 10 mg/Kg) and anti-N1 (10 mg/Kg). Both IgG and anti-N1 were delivered intraperitoneally every other day. This regimen led to a significant delay in tumor growth in both lines (Fig. 4A, and Suppl. Figure 5 A), and importantly, altered the TME. We observed reduced monocytic MDSCs (CD11b^+^; Ly6C^hi^; ly6G^−^) and Tregs (CD4^+^/FoxP3^+^/CD25^hi^); and increased CD4^+^ and CD8^+^ T cells, resulting in a significant decrease of the Tregs/CD8 ratio (Fig. 4B and Suppl. Figure 5B). Polymorphonuclear MDSCs did not change (not shown). These data recapitulate our previous observations in which Notch1 was inhibited via RNAi [9]. The gating strategy is shown in suppl. Figure 6. Of note, while anti-N1 did not affect the GI tract (Fig. 4C, D), as shown by maintenance of weight throughout the experiment and similar levels of proliferating cells in the intestinal crypts in both controls and anti-N1 treated animals, DBZ treated mice demonstrated a significantly reduced weight after a two week treatment, and a significantly reduced number of Ki67 positive cells in the crypts, supporting a more targeted inhibition of Notch1 as a safer choice to avoid GI side effects which are common with GSIs (Suppl. Figure 7).

Additionally, even though DBZ (10umol/Kg) delivered I.P. for three days followed by four days holiday period [16], did reduce tumor growth (Suppl. Figure 8 A), it caused overall immunosuppression in the TME of melanomas. We observed depletion of MDSCs and Tregs (Suppl. Figure 8B, C), but also of CD4^+^ and CD8^+^ T cells (Suppl. Figure 8D, E), in the TME and in the spleen. The Tregs/CD8 ratio in the TME did not change as we observed in tumors depleted of Notch1 only (Suppl. Figure 8I), likely because both the Tregs and the CD8^+^ populations were depleted simultaneously.

Together, these data indicate anti-N1 is selective and non-toxic to normal cells; it does not affect the GI tract; and it delivers anti-tumor activity without the immunosuppressive property of GSIs, thus supporting its use as a safer anti-melanoma approach than pan-Notch inhibition.

### Anti-N1 favors CD8 ^+^ T cell cytotoxicity and anti-tumor activity

**Fig. 5:**
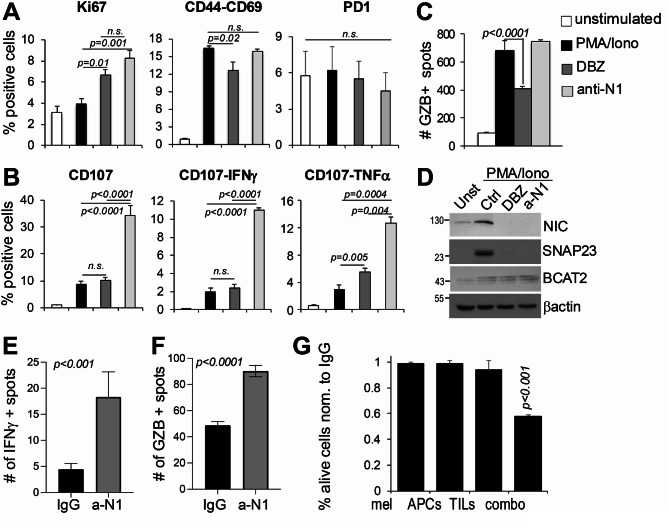
Anti-N1 promotes CD8^+^ T cytotoxicity. **A, B)** Flow cytometry for markers of cell growth (Ki67), activity (CD44/CD69), exhaustion (PD-1), degranulation (CD107a), IFNγ and TNFα in CD8^+^ T cells unstimulated or stimulated with PMA (50ng/ml) + ionomycin (500ng/ml) for 4 h in vitro. **C)** Granzyme B^+^ CD8 T cells treated as in A-B. Dots were counted by ELISpot. **D)** Expression of active Notch1 (NIC), the Notch1 selective target SNAP23 and the Notch2 selective target BCAT2 in CD8^+^ T cells. Data are the mean of 3 independent experiments. **E**,** F)** IFNγ and GZB ELISpot data from treated tumors. Data are the mean of two independent experiments each containing 5 tumors. P values were calculated by the Student’s t test. **G)** Naïve YUMM2.1 melanoma cells were co-cultured with TILs extracted from YUMM2.1 tumors treated with IgG or anti-N1, and APCs at a 5:1 ratio, then treated with 10ug/ml anti-N1 for an additional 12 h. Cells were then harvested for flow cytometry. Data are the % of alive cells in the anti-N1 group normalized to control (IgG), which was set at 1 for all treatments. Data are the mean of three independent experiments. Combo = melanoma + TILs + APCs

Given the increase in CD8^+^ T cells in the TME, we sought to investigate whether the anti-tumor effect observed in mice treated with anti-N1 was due to increased cytotoxicity of these effector cells. First, CD8^+^ T cells, obtained from naïve C57Bl/6 spleens, were stimulated with PMA/ionomycin in vitro then treated with either DBZ or anti-N1. Interestingly, both DBZ and anti-N1 increased CD8^+^ T cell proliferation (% Ki67^+^ cells), however, while DBZ decreased active CD8^+^ T cells (% CD44^+^/CD69^+^ cells), anti-N1 did not. No changes in the expression of PD-1 were observed with either DBZ or anti-N1, however, the antibody increased the frequency of CD107a^+^ degranulated cells as well as CD107-IFNγ and CD107-TNFα positive cells (Fig. 5A, B). Granzyme B positivity was unchanged by anti-N1 but significantly diminished by DBZ (ELISpot assay, Fig. 5C). Of note anti-N1 reduced Notch1 activity in stimulated CD8^+^ T cells, as shown by lower levels of both activated Notch1 (NIC) and of SNAP23; but it did not affect BCAT2, a selective target of Notch2, indicating Notch2 is likely active in CD8^+^ T cells (Fig. 5D). These data support that the selective blockade of Notch1 in CD8^+^ T cells increases their cytotoxic ability, which is likely to exert anti-tumor activity.

To test this notion ex vivo, TILs (tumor infiltrating lymphocytes – CD45^+^) were extracted from YUMM2.1 melanoma tumors (*n* = 5) that were treated for 14 days with either IgG or anti-N1 (10 mg/Kg, every other day). TILs were then cultured overnight in the presence of anti-N1 or control IgG, and the production of IFNγ and granzyme B assessed by ELISpot. Both were significantly produced by TILs from mice treated with anti-N1 (Fig. 5E, F and Suppl. Figure 9), indicating these cells are likely cytotoxic. We therefore tested the anti-tumor activity of TILs ex vivo. TILs extracted from YUMM2.1 tumors treated as above, were cultured with APCs and naïve YUMM2.1 melanoma cells, then anti-N1 was added for 12 h. At this concentration (10ug/ml) and duration, the antibody does not kill melanoma cells directly (direct cell death is significant only after 96 h at this concentration, or 72 h at 25ug/ml – Fig. 3), however, aided TILs mediated melanoma cell death, as shown by a 40% reduction in viable melanoma cells in the co-culture, compared to TILs extracted from IgG treated tumors (Fig. 5G, Suppl. Figure 10 - data are normalized to IgG treated cells, set at 1 as reference).

Overall, these data support the selective inhibition of Notch1 to both bypass the gastrointestinal side effects and global immunosuppression associated with pan-Notch inhibition and most importantly, to maintain an anti-tumor CD8^+^ T cell population needed to kill the tumor cells.

### Anti-Notch1 boosts anti-PD-1 treatment

**Fig. 6:**
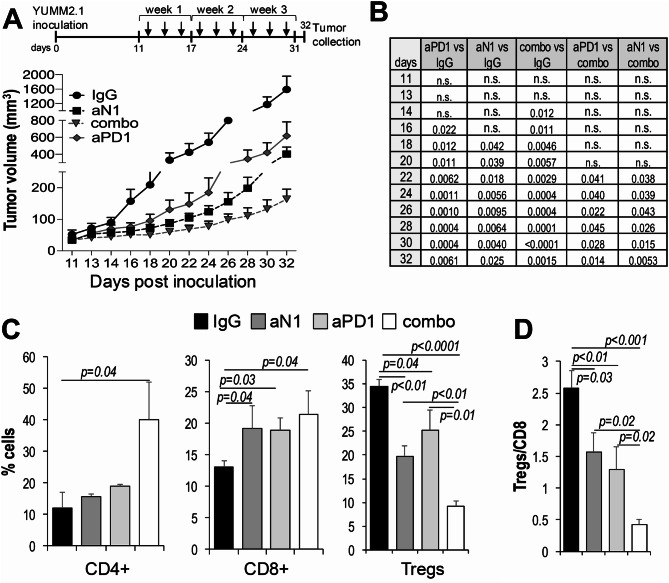
Anti-N1 boosts anti-PD-1 treatment. **(A)** Tumor growth of YUMM2.1 cells inoculated s.c. into C57BL/6 mice. Treatment with IgG control (10 mg/Kg), anti-N1 (10 mg/Kg) or anti PD-1 (100ug/mouse) started at day 11 post inoculation, when tumors reached an average volume of 100-150mm^3^. The Y axis, representing tumor growth, has been sectioned into three lines to better show the separation in growth among treatments and between treatments and control. **(B)** Student’s t test for each time point. n.s.= not significant. **(C)** % of CD4^+^ T cells, CD8 + T cells, and Tregs in the TME of each treatment group. **(D)** Tregs/CD8 ratio. *N* = 20 per group. This experiment was performed twice, and data combined, with *n* = 10 per group, per experiment. Significance was calculated by the Student’s t test

In view of the anti-tumor activity of anti-N1, its ability to stimulate an inflamed TME with increased cytotoxic CD8^+^ T cells, and its safety, we sought to determine whether anti-N1 could boost the anti-tumor effects of anti-PD-1. YUMM2.1 were chosen as model system as they are sensitive to both anti-PD-1 [26] and anti-N1. Tumors were allowed to grow to approximately 100-150mm^3^, and animals were then divided into four groups of treatment, containing mice with comparable tumor volumes: IgG control (10 mg/Kg); anti-N1 (10 mg/Kg); anti-PD-1 (100 µg/mouse [27]); combo (aN1 + a-PD-1), delivered I.P. every other day. Both a-N1 and a-PD-1 exerted anti-tumor activity, with the combination further delaying tumor growth (Fig. 6A). A significant separation in the growth curves of the combination therapy compared to either monotherapy, was apparent at day 22 post inoculation (Fig. 6B), which falls into the second week of treatment, and continued until the end time point (day 32), when animals were euthanized, and tumors collected for flow cytometry. Analysis of the TME revealed a trend in CD4^+^ cells increase in the monotherapy, albeit not significant (*p* = 0.07), however, the combination therapy boosted the percentage of CD4^+^ T cells in the TME. A significant increase in CD8^+^ T cells was observed in all treatment groups, however, given that the Tregs population was significantly inhibited in both monotherapies, and more so in the combination, the resultant Tregs/CD8 ratio was significantly reduced in all treatment groups, especially in the combination.

Notably, DBZ, while exerting a slight anti-tumor activity in YUMM2.1, it did not boost the anti-tumor activity of anti-PD-1 (Suppl. Figure 11).

Overall, these data demonstrate that selective inhibition of Notch1 exerts anti-melanoma therapy with immunomodulatory activity that boosts ICI treatment with anti-PD-1.

## Discussion

Although immunotherapy with combination anti-PD-1 and anti-CTLA-4 delivers durable responses in melanoma patients, half of the patient population demonstrates resistance, either intrinsically or acquired. Several mechanisms of resistance have been identified. High expression of PD-L1 and 2 for example, counteracts CD8^+^ T cell activation and tumoricidal function. Melanomas can also selectively attract immune inhibitory cells such as Tregs and MDSCs; or show low antigen presentation and recognition due to low mutational burden and loss of MHC class I. Melanoma may also present insufficient mature dendritic cells (DCs) in the TME that can limit the generation of potent T cell responses; and have mutations such as in the JAK/STAT pathway that decrease IFNγ signaling sensitivity (reviewed in [41, 42]). We propose elevated Notch1 signaling activity is also a mechanism of resistance to melanoma immunotherapy.

We show that elevated Notch1 and Notch canonical target genes, which act as readout of Notch activity, are associated with a non-inflamed TME, and that elevated expression of Notch activity associates with non-responders to ICIs. Selective Notch1 inhibition with a novel neutralizing monoclonal antibody developed in our laboratory not only exerts anti-tumor activity as a monotherapy, but importantly, boosts the therapeutic effects of anti-PD-1. Mechanistically, we find inhibition of Notch1 increases CD8^+^ T cell degranulation and IFNγ and Granzyme B production both in vitro and in vivo, leading to melanoma cell killing.

These data support previous findings from our group of a role of Notch1 in promoting a non-inflamed melanoma TME [9], and, importantly, provide a novel translational means to selectively target Notch1 in melanoma. This is quite important because by inhibiting Notch1 only, Notch2 is left unaffected and able to maintain GI homeostasis, as well as the cytotoxic properties of CD8^+^ T cells. This cannot be achieved with GSIs. Indeed, we show that the use of the GSI dibenzazepine (DBZ) in a syngeneic melanoma model results in the reduction of both pro-tumorigenic (Tregs, MDSCs) and anti-tumorigenic cells (CD4^+^ and CD8^+^ T cells), essentially leading to overall immunosuppression; and requires staggered delivery of the drug, with a three-day treatment followed by a four-day holiday period to allow the animals to regain weight, as previously demonstrated [16], which may negatively affect the GSI anti-tumor properties. On the other hand, anti-N1 was safe, allowing mice to maintain and even slightly gain weight over time, and demonstrating normal intestines, indicating lack of GI toxicities. Importantly, anti-N1 promoted CD8^+^ T cells cytotoxicity. Even in the combination therapy anti-N1/anti-PD1, animals maintained and gained weight over time (not shown), again suggesting lack of GI side effects. This is important as ICI combination therapies such as anti-PD-1/anti-CTLA-4, while it leads to prolonged survival in melanoma patients [1], are often accompanied by severe immunogenic adverse events, mostly to the GI tract, skin, liver, lungs, that can interrupt or even suspend the therapy, if not manageable [43].

In summary, we show anti-N1 is well tolerated in mice harboring melanoma tumors; it does not cause GI-associated side effects alone or in combination with anti-PD-1; and importantly, exerts significant anti-tumor activity as monotherapy, partly by increasing tumor CD8^+^ T cell cytotoxicity, explaining why anti-N1 boosts anti-PD-1 therapy. Future studies will investigate the potential of anti-N1 in combination with other ICIs and the possible immunogenic adverse events and will further define the anti-tumor mechanisms of anti-N1. In conclusion, we propose selective Notch1 inhibition with our anti-N1 antibody as a novel, effective anti-melanoma therapy, particularly in combination with immunotherapy.

## Electronic supplementary material

Below is the link to the electronic supplementary material.

Supplementary Material 1

## Data Availability

The data generated during the current study are available from the corresponding author on reasonable request.

## References

[1] Wolchok JD, Chiarion-Sileni V, Gonzalez R, Grob JJ, Rutkowski P, Lao CD, Cowey CL, Schadendorf D, Wagstaff J, Dummer R, et al. Long-term outcomes with Nivolumab Plus Ipilimumab or Nivolumab alone Versus Ipilimumab in patients with Advanced Melanoma. J Clin Oncol. 2022;40(2):127–37. 34818112 10.1200/JCO.21.02229 PMC8718224

[2] Ribas A, Hamid O, Daud A, Hodi FS, Wolchok JD, Kefford R, Joshua AM, Patnaik A, Hwu WJ, Weber JS, et al. Association of Pembrolizumab with Tumor Response and Survival among patients with Advanced Melanoma. JAMA. 2016;315(15):1600–9. 27092830 10.1001/jama.2016.4059

[3] Spranger S, Bao R, Gajewski TF. Melanoma-intrinsic beta-catenin signalling prevents anti-tumour immunity. Nature. 2015;523(7559):231–5. 25970248 10.1038/nature14404

[4] Spranger S, Dai D, Horton B, Gajewski TF. Tumor-residing Batf3 dendritic cells are required for effector T cell trafficking and adoptive T cell therapy. Cancer Cell. 2017;31(5):711–e723714. 28486109 10.1016/j.ccell.2017.04.003 PMC5650691

[5] Peng W, Chen JQ, Liu C, Malu S, Creasy C, Tetzlaff MT, Xu C, McKenzie JA, Zhang C, Liang X, et al. Loss of PTEN promotes resistance to T cell-mediated immunotherapy. Cancer Discov. 2016;6(2):202–16. 26645196 10.1158/2159-8290.CD-15-0283 PMC4744499

[6] Chen DS, Mellman I. Elements of cancer immunity and the cancer-immune set point. Nature. 2017;541(7637):321–30. 28102259 10.1038/nature21349

[7] Griss J, Bauer W, Wagner C, Simon M, Chen M, Grabmeier-Pfistershammer K, Maurer-Granofszky M, Roka F, Penz T, Bock C, et al. B cells sustain inflammation and predict response to immune checkpoint blockade in human melanoma. Nat Commun. 2019;10(1):4186. 31519915 10.1038/s41467-019-12160-2 PMC6744450

[8] Ayers M, Lunceford J, Nebozhyn M, Murphy E, Loboda A, Kaufman DR, Albright A, Cheng JD, Kang SP, Shankaran V, et al. IFN-gamma-related mRNA profile predicts clinical response to PD-1 blockade. J Clin Invest. 2017;127(8):2930–40. 28650338 10.1172/JCI91190 PMC5531419

[9] Qiu H, Zmina PM, Huang AY, Askew D, Bedogni B. Inhibiting Notch1 enhances immunotherapy efficacy in melanoma by preventing Notch1 dependent immune suppressive properties. Cancer Lett. 2018;434:144–51. 30036609 10.1016/j.canlet.2018.07.024 PMC7185871

[10] Osawa M, Fisher DE. Notch and melanocytes: diverse outcomes from a single signal. J Invest Dermatol. 2008;128(11):2571–4. 18927539 10.1038/jid.2008.289

[11] Bedogni B, Warneke JA, Nickoloff BJ, Giaccia AJ, Powell MB. Notch1 is an effector of akt and hypoxia in melanoma development. J Clin Invest. 2008;118(11):3660–70. 18924608 10.1172/JCI36157 PMC2567838

[12] Zhang K, Wong P, Zhang L, Jacobs B, Borden EC, Aster JC, Bedogni B. A Notch1-neuregulin1 autocrine signaling loop contributes to melanoma growth. Oncogene. 2012;31(43):4609–18. 22249266 10.1038/onc.2011.606 PMC4201386

[13] Balint K, Xiao M, Pinnix CC, Soma A, Veres I, Juhasz I, Brown EJ, Capobianco AJ, Herlyn M, Liu ZJ. Activation of Notch1 signaling is required for beta-catenin-mediated human primary melanoma progression. J Clin Invest. 2005;115(11):3166–76. 16239965 10.1172/JCI25001 PMC1257536

[14] Liu ZJ, Xiao M, Balint K, Smalley KS, Brafford P, Qiu R, Pinnix CC, Li X, Herlyn M. Notch1 signaling promotes primary melanoma progression by activating mitogen-activated protein kinase/phosphatidylinositol 3-kinase-akt pathways and up-regulating N-cadherin expression. Cancer Res. 2006;66(8):4182–90. 16618740 10.1158/0008-5472.CAN-05-3589

[15] Pinnix CC, Lee JT, Liu ZJ, McDaid R, Balint K, Beverly LJ, Brafford PA, Xiao M, Himes B, Zabierowski SE, et al. Active Notch1 confers a transformed phenotype to primary human melanocytes. Cancer Res. 2009;69(13):5312–20. 19549918 10.1158/0008-5472.CAN-08-3767 PMC2755513

[16] Zhang K, Wong P, Salvaggio C, Salhi A, Osman I, Bedogni B. Synchronized targeting of Notch and ERBB Signaling suppresses Melanoma Tumor Growth through Inhibition of Notch1 and ERBB3. J Invest Dermatol. 2016;136(2):464–72. 26967479 10.1016/j.jid.2015.11.006 PMC4789778

[17] McCaw TR, Inga E, Chen H, Jaskula-Sztul R, Dudeja V, Bibb JA, Ren B, Rose JB. Gamma secretase inhibitors in Cancer: a current perspective on clinical performance. Oncologist. 2021;26(4):e608–21. 33284507 10.1002/onco.13627 PMC8018325

[18] Sugimoto K, Maekawa Y, Kitamura A, Nishida J, Koyanagi A, Yagita H, Kojima H, Chiba S, Shimada M, Yasutomo K. Notch2 signaling is required for potent antitumor immunity in vivo. J Immunol. 2010;184(9):4673–8. 20351182 10.4049/jimmunol.0903661

[19] Dai K, Huang L, Huang YB, Chen ZB, Yang LH, Jiang YA. 1810011o10 Rik inhibits the Antitumor Effect of Intratumoral CD8(+) T cells through suppression of Notch2 pathway in a murine Hepatocellular Carcinoma Model. Front Immunol. 2017;8:320. 28382040 10.3389/fimmu.2017.00320 PMC5360711

[20] Riccio O, van Gijn ME, Bezdek AC, Pellegrinet L, van Es JH, Zimber-Strobl U, Strobl LJ, Honjo T, Clevers H, Radtke F. Loss of intestinal crypt progenitor cells owing to inactivation of both Notch1 and Notch2 is accompanied by derepression of CDK inhibitors p27Kip1 and p57Kip2. EMBO Rep. 2008;9(4):377–83. 18274550 10.1038/embor.2008.7 PMC2288761

[21] Deangelo D, Stone R, Silverman L, Stock W, Attar E, Fearen I, Dallob A, Matthews C, Stone J, Freedman S et al. A phase I clinical trial of the notch inhibitor MK-0752 in patients with T-cell acute lymphoblastic leukemia/lymphoma (T-ALL) and other leukemias. J Clin Oncol 2006(24):6585.

[22] Searfoss GH, Jordan WH, Calligaro DO, Galbreath EJ, Schirtzinger LM, Berridge BR, Gao H, Higgins MA, May PC, Ryan TP. Adipsin, a biomarker of gastrointestinal toxicity mediated by a functional gamma-secretase inhibitor. J Biol Chem. 2003;278(46):46107–16. 12949072 10.1074/jbc.M307757200

[23] Li T, Wen H, Brayton C, Das P, Smithson LA, Fauq A, Fan X, Crain BJ, Price DL, Golde TE, et al. Epidermal growth factor receptor and notch pathways participate in the tumor suppressor function of gamma-secretase. J Biol Chem. 2007;282(44):32264–73. 17827153 10.1074/jbc.M703649200

[24] Marusak C, Thakur V, Li Y, Freitas JT, Zmina P, Thakur VS, Chang M, Gao M, Tan J, Xiao M et al. Targeting Extracellular Matrix Remodeling restores BRAF inhibitor sensitivity in BRAFi resistant melanoma. Clin Cancer Res 2020;26(22):6039–6050. 10.1158/1078-0432.CCR-19-2773. 10.1158/1078-0432.CCR-19-2773 PMC7669662 32820016

[25] Meeth K, Wang JX, Micevic G, Damsky W, Bosenberg MW. The YUMM lines: a series of congenic mouse melanoma cell lines with defined genetic alterations. Pigment Cell Melanoma Res. 2016;29(5):590–7. 27287723 10.1111/pcmr.12498 PMC5331933

[26] Homet Moreno B, Zaretsky JM, Garcia-Diaz A, Tsoi J, Parisi G, Robert L, Meeth K, Ndoye A, Bosenberg M, Weeraratna AT, et al. Response to programmed cell Death-1 blockade in a murine Melanoma Syngeneic Model requires Costimulation, CD4, and CD8 T cells. Cancer Immunol Res. 2016;4(10):845–57. 27589875 10.1158/2326-6066.CIR-16-0060 PMC5050168

[27] Wei Z, Zhang X, Yong T, Bie N, Zhan G, Li X, Liang Q, Li J, Yu J, Huang G, et al. Boosting anti-PD-1 therapy with metformin-loaded macrophage-derived microparticles. Nat Commun. 2021;12(1):440. 33469052 10.1038/s41467-020-20723-x PMC7815730

[28] Crowe AR, Yue W. Updated: semi-quantitative determination of protein expression using immunohistochemistry staining and analysis. Bio Protoc 2023, 2(13). 10.21769/BioProtoc.4610 PMC9901447 36789249

[29] Li T, Fu J, Zeng Z, Cohen D, Li J, Chen Q, Li B, Liu XS. TIMER2.0 for analysis of tumor-infiltrating immune cells. Nucleic Acids Res. 2020;48(W1):W509–14. 32442275 10.1093/nar/gkaa407 PMC7319575

[30] Hugo W, Zaretsky JM, Sun L, Song C, Moreno BH, Hu-Lieskovan S, Berent-Maoz B, Pang J, Chmielowski B, Cherry G, et al. Genomic and transcriptomic features of response to Anti-PD-1 therapy in metastatic melanoma. Cell. 2016;165(1):35–44. 26997480 10.1016/j.cell.2016.02.065 PMC4808437

[31] Amato CM, Hintzsche JD, Wells K, Applegate A, Gorden NT, Vorwald VM, Tobin RP, Nassar K, Shellman YG, Kim J et al. Pre-treatment Mutational and Transcriptomic Landscape of responding metastatic melanoma patients to Anti-PD1 immunotherapy. Cancers (Basel) 2020, 12(7). 10.3390/cancers12071943 PMC7409244 32708981

[32] Riaz N, Havel JJ, Makarov V, Desrichard A, Urba WJ, Sims JS, Hodi FS, Martin-Algarra S, Mandal R, Sharfman WH, et al. Tumor and Microenvironment Evolution during Immunotherapy with Nivolumab. Cell. 2017;171(4):934–e949916. 29033130 10.1016/j.cell.2017.09.028 PMC5685550

[33] Gordon WR, Roy M, Vardar-Ulu D, Garfinkel M, Mansour MR, Aster JC, Blacklow SC. Structure of the Notch1-negative regulatory region: implications for normal activation and pathogenic signaling in T-ALL. Blood. 2009;113(18):4381–90. 19075186 10.1182/blood-2008-08-174748 PMC2676092

[34] Gordon WR, Arnett KL, Blacklow SC. The molecular logic of notch signaling–a structural and biochemical perspective. J Cell Sci. 2008;121(Pt 19):3109–19. 18799787 10.1242/jcs.035683 PMC2696053

[35] Matys V, Fricke E, Geffers R, Gossling E, Haubrock M, Hehl R, Hornischer K, Karas D, Kel AE, Kel-Margoulis OV, et al. TRANSFAC: transcriptional regulation, from patterns to profiles. Nucleic Acids Res. 2003;31(1):374–8. 12520026 10.1093/nar/gkg108 PMC165555

[36] Matys V, Kel-Margoulis OV, Fricke E, Liebich I, Land S, Barre-Dirrie A, Reuter I, Chekmenev D, Krull M, Hornischer K, et al. TRANSFAC and its module TRANSCompel: transcriptional gene regulation in eukaryotes. Nucleic Acids Res. 2006;34(Database issue):D108–110. 16381825 10.1093/nar/gkj143 PMC1347505

[37] Ferrando AA. The role of NOTCH1 signaling in T-ALL. Hematol Am Soc Hematol Educ Program 2009:353–61. 10.1182/asheducation-2009.1.353 PMC2847371 20008221

[38] Agnusdei V, Minuzzo S, Frasson C, Grassi A, Axelrod F, Satyal S, Gurney A, Hoey T, Seganfreddo E, Basso G, et al. Therapeutic antibody targeting of Notch1 in T-acute lymphoblastic leukemia xenografts. Leukemia. 2014;28(2):278–88. 23774673 10.1038/leu.2013.183

[39] Ferrarotto R, Eckhardt G, Patnaik A, LoRusso P, Faoro L, Heymach JV, Kapoun AM, Xu L, Munster P. A phase I dose-escalation and dose-expansion study of brontictuzumab in subjects with selected solid tumors. Ann Oncol. 2018;29(7):1561–8. 29726923 10.1093/annonc/mdy171

[40] Aste-Amezaga M, Zhang N, Lineberger JE, Arnold BA, Toner TJ, Gu M, Huang L, Vitelli S, Vo KT, Haytko P, et al. Characterization of Notch1 antibodies that inhibit signaling of both normal and mutated Notch1 receptors. PLoS ONE. 2010;5(2):e9094. 20161710 10.1371/journal.pone.0009094 PMC2817004

[41] Gide TN, Wilmott JS, Scolyer RA, Long GV. Primary and Acquired Resistance to Immune checkpoint inhibitors in metastatic melanoma. Clin Cancer Res. 2018;24(6):1260–70. 29127120 10.1158/1078-0432.CCR-17-2267

[42] Huang AC, Zappasodi R. A decade of checkpoint blockade immunotherapy in melanoma: understanding the molecular basis for immune sensitivity and resistance. Nat Immunol. 2022;23(5):660–70. 35241833 10.1038/s41590-022-01141-1 PMC9106900

[43] Schneider BJ, Naidoo J, Santomasso BD, Lacchetti C, Adkins S, Anadkat M, Atkins MB, Brassil KJ, Caterino JM, Chau I, et al. Management of Immune-related adverse events in patients treated with Immune checkpoint inhibitor therapy: ASCO Guideline Update. J Clin Oncol. 2021;39(36):4073–126. 34724392 10.1200/JCO.21.01440

