# Supplementary material for: Notch1 blockade by a novel, selective anti-Notch1 neutralizing antibody improves immunotherapy efficacy in melanoma by promoting an inflamed TME
Source: J Exp Clin Cancer Res. 2024 Nov 4;43:295. doi: 10.1186/s13046-024-03214-5 (PMC11533310; doi:10.1186/s13046-024-03214-5)
Supplement: Supplementary file 1 — Supplementary Material 1 [file 13046_2024_3214_MOESM1_ESM.pdf]

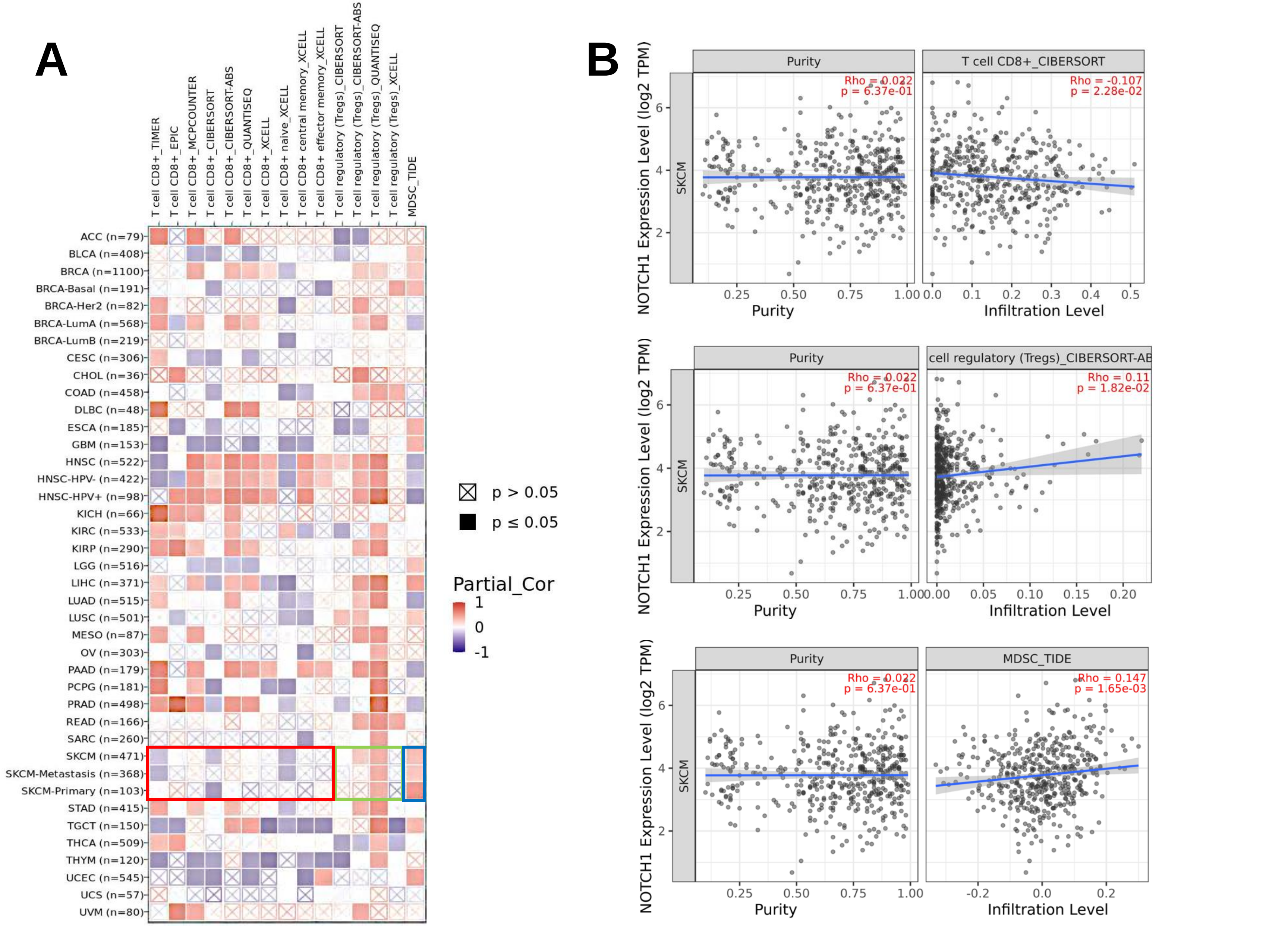

Suppl. Fig. 1: Timer 2.0 immune infiltration in TCGA tumors correlated with Notch1. A) complete heat map comprising all TCGA tumor types. Melanoma: SKCM. Red square: CD8+ T cells; Green square: Tregs; blue square: MDSCs. **B)** representative correlation between Notch1 and CD8+ T cells, Tregs or MDSCs infiltrate from the CIBERSORT and TIDE algorithms. Spearman's correlation, $p<0.05$.

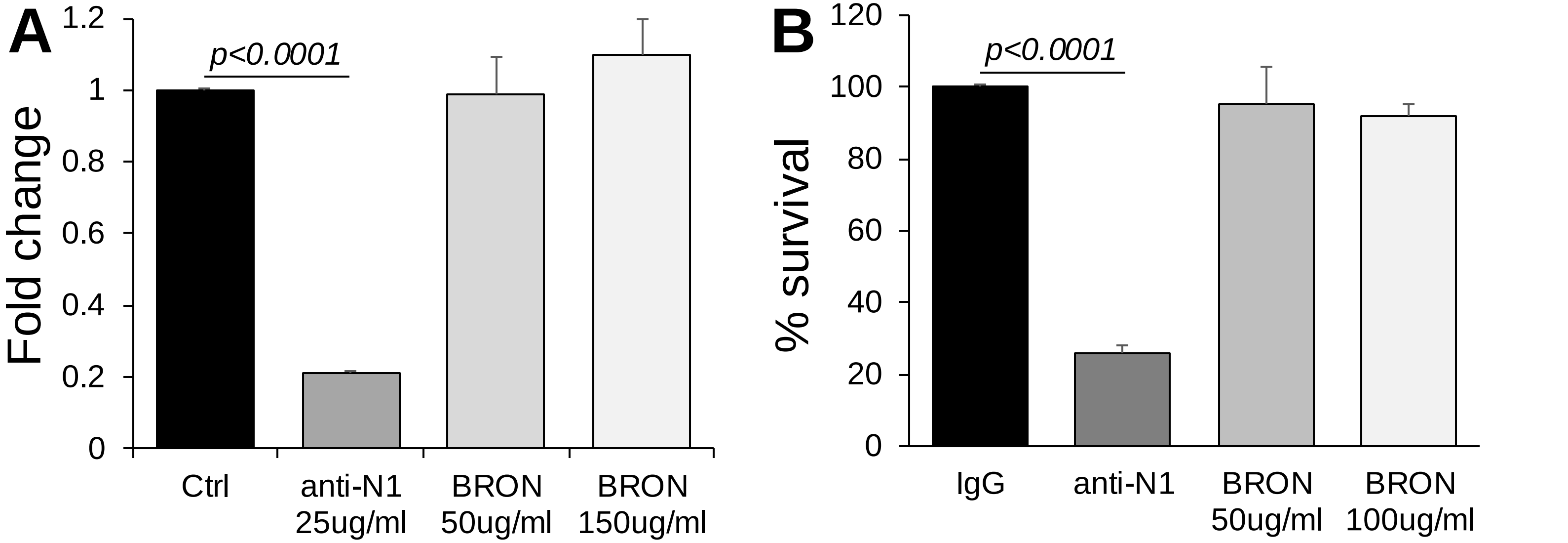

Suppl. Fig. 2: Brontictuzumab does not affect Notch1 activity or cell survival. A) qRT-PCR of SNAP23 in YUMM2.1 cells treated for three days with IgG control (100ug/ml) or anti-N1 (25ug/ml) or brontictuzumab (BRON – 50, 100 ug/ml). **B)** % survival of the cells in A, normalized to IgG control, set at 100%. $p<0.0001$, Student's t test.

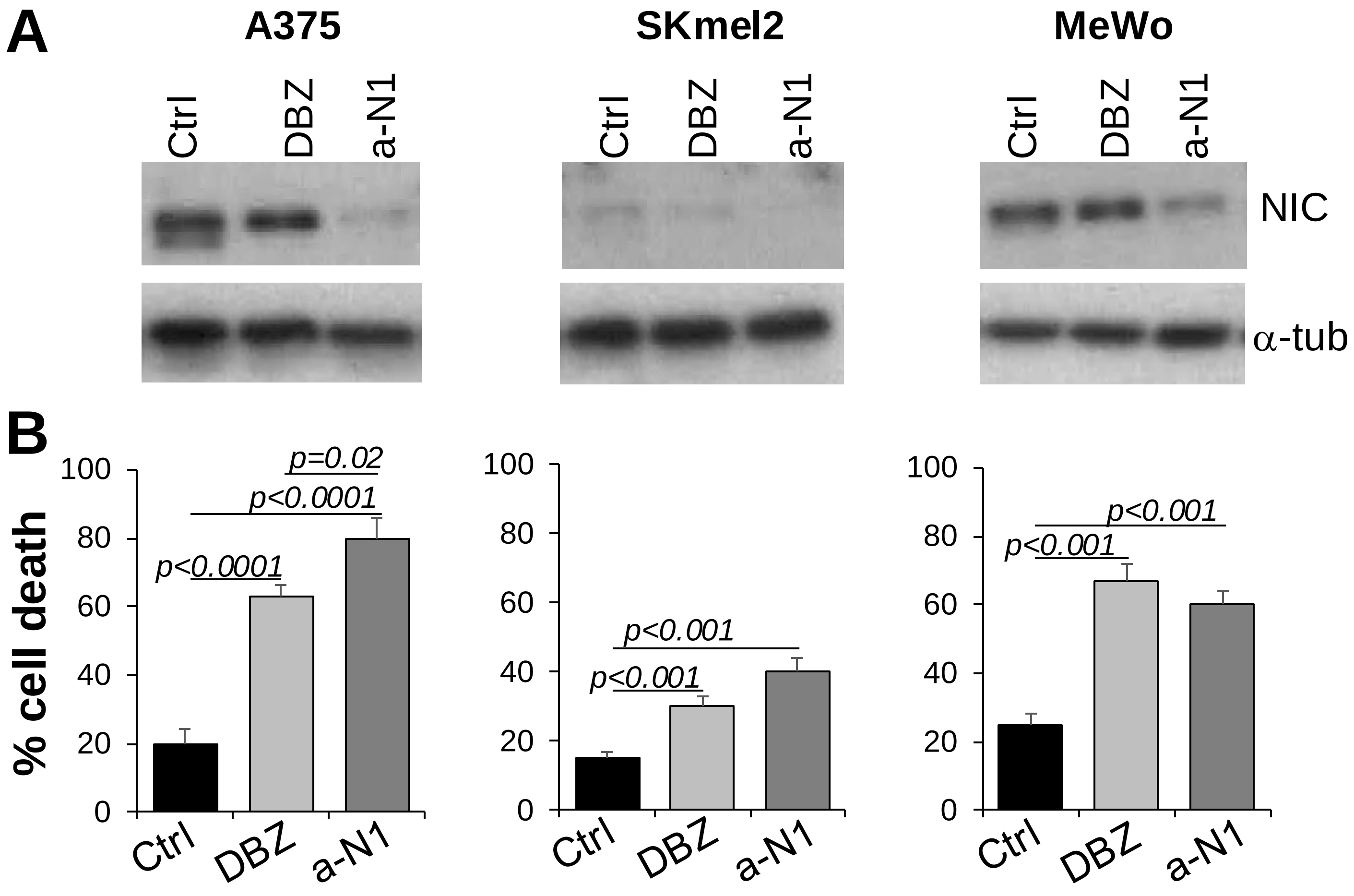

Suppl. Fig. 3: anti-N1 reduces Notch1 activation and causes cell death in human melanoma cells: A) Notch1-NIC expression in A375, SKmel2 and MeWo cells. α-tubulin was used as loading control. **B)** % cell death of the cells in A (Trypan blue exclusion assay), after a three-day treatment with either IgG/DMSO, the GSI DBZ (10uM) or anti-N1 (25ug/ml). Values are the mean of two independent experiments each performed in triplicate. P values were determined by the Student's *t* test.

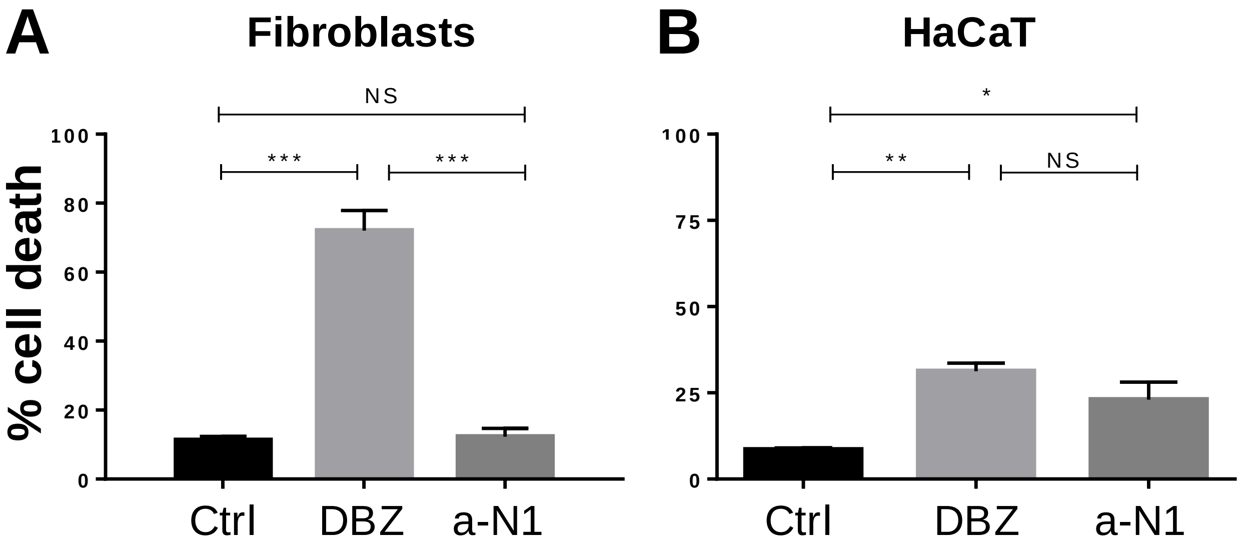

Suppl. Fig. 4: anti-N1 does not affect normal human fibroblast and HaCaT cells; A-B) % dead cells (Trypan blue exclusion assay) for normal human fibroblasts and HaCaT cells after a three-day treatment with either the GSI DBZ (10uM) or anti-N1 (25ug/ml). $p<0.05$, Student’s t test.

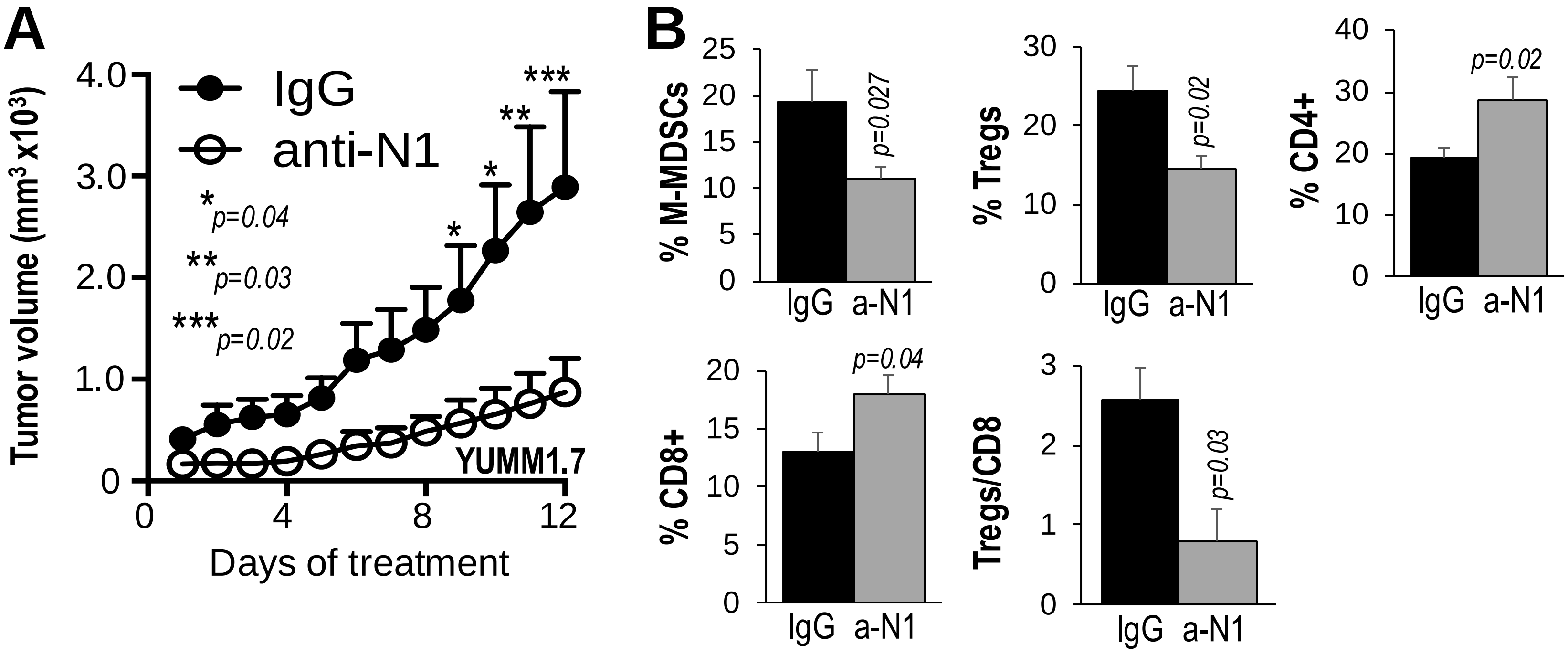

Suppl. Fig. 5: A) Growth rates of YUMM1.7 tumors treated with IgG or anti-N1 (10mg/Kg) every other day. n=10 per group. **B)** % M-MDSCs (CD11b+; Ly6C^{hi}; ly6G^{-} = monocytic), Tregs (CD4+/FoxP3+), CD4+ T cells, CD8+ T cells, in YUMM1.7 tumors from A. The Tregs/CD8 ratio was calculated by dividing the absolute number of CD4+/FoxP3+ and CD8+ T cells in tumors. Absolute numbers were obtained by normalizing the number of cells detected by Flow cytometry to the tumor mass. Data are the mean of two independent experiments. P values were calculated by the Student’s t test.

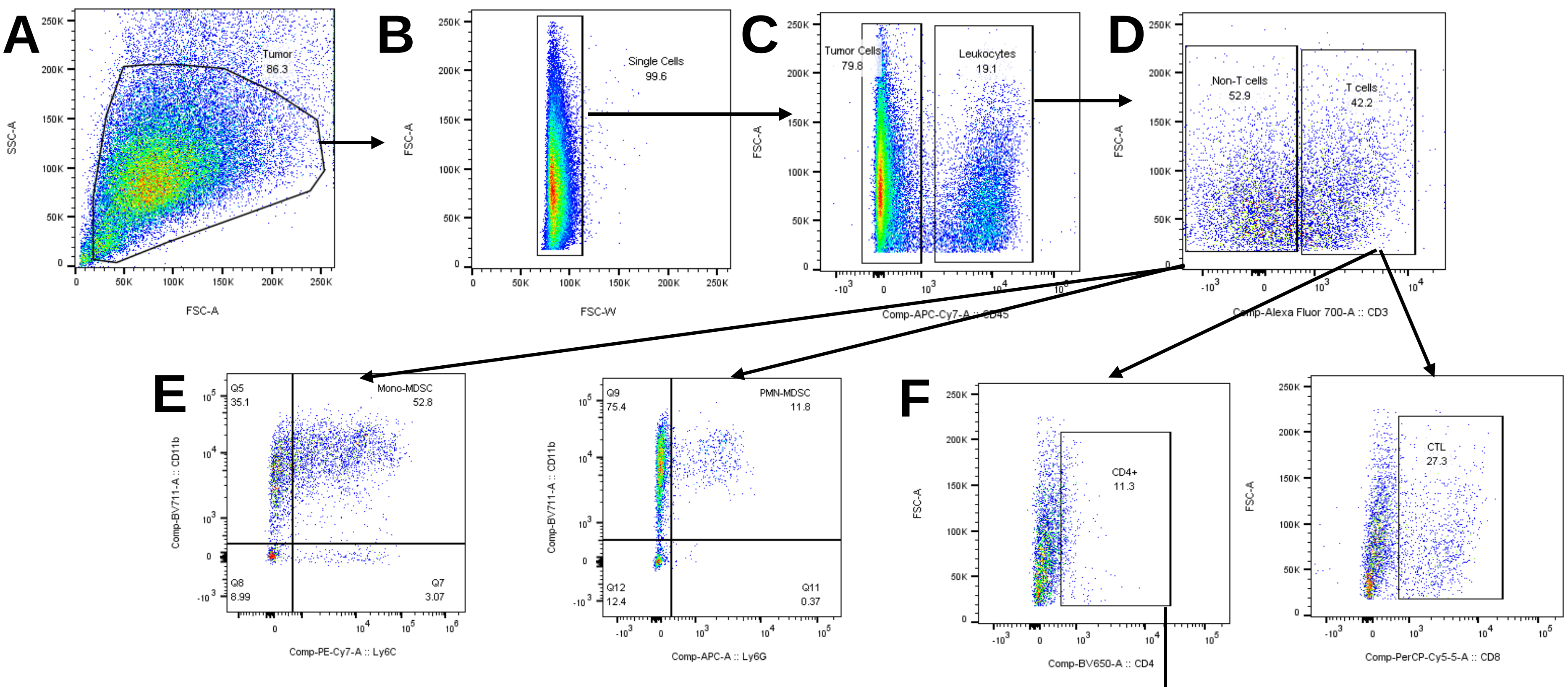

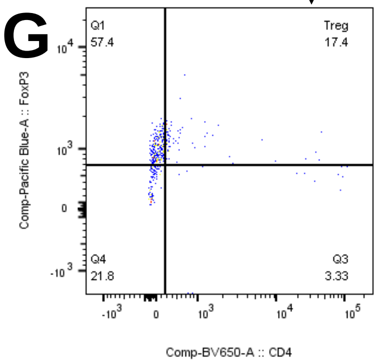

Suppl. Fig. 6: Gating strategy: A) Forward (FSC-A) and side (SSC-A) scatter highlight cancer cells. **B)** isolation of singlets cells vs doublets. **C)** CD45+ cells (leukocytes). **D)** CD3+ (T-cells) and (-) (non T-cells). **E)** CD3(-)/CD11b+/LyC+ (monocytic MDSCs) and CD3(-)/CD11b+/LyG+ (polymorphonuclear MDSCs). **F)** CD3+/CD4+ and CD3+/CD8+. **G)** CD3+/CD4+/FoxP3+ (Tregs).

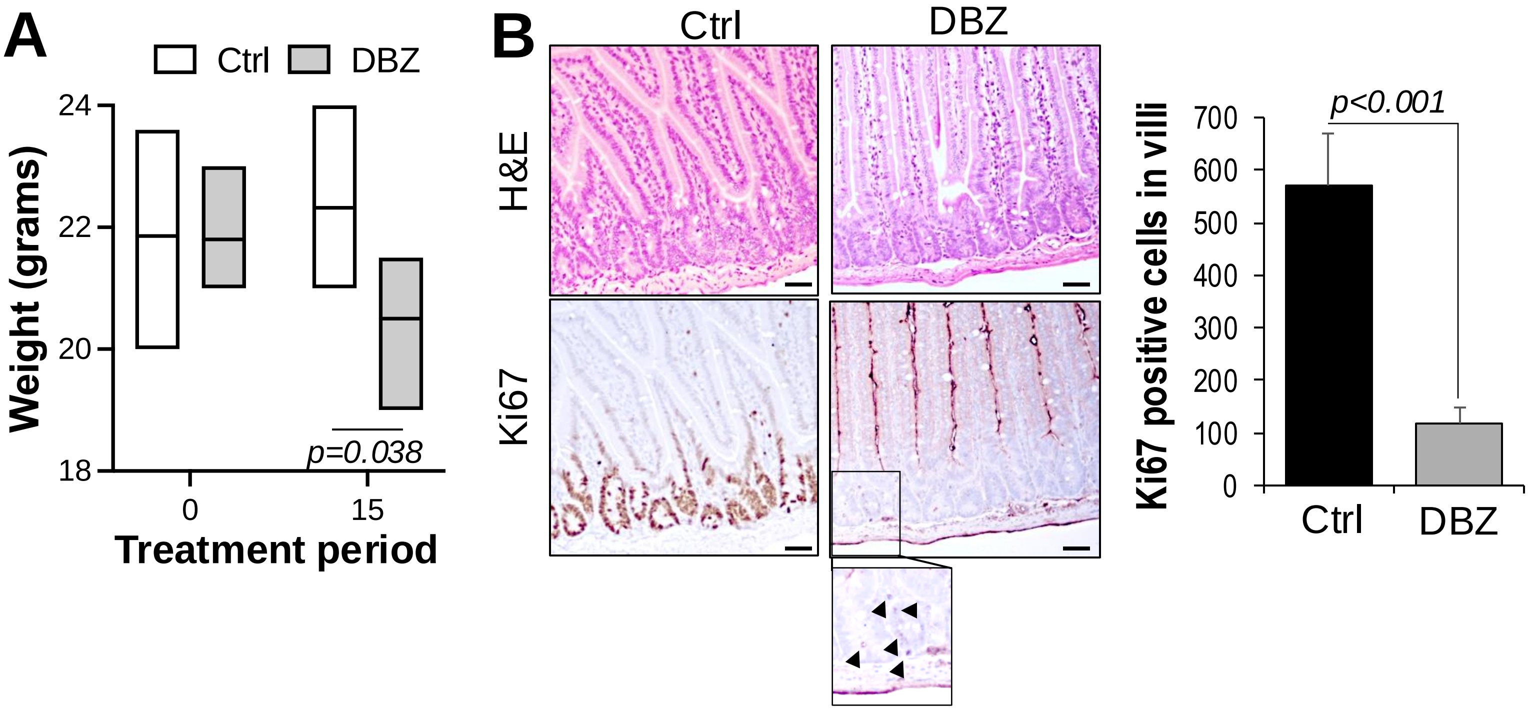

Suppl. Fig. 7: A) C57B/6 mice were treated for two weeks with DBZ (10umol/Kg) or DMSO (Ctrl), every other day. Animal weigh was measured at time 0 prior to treatment initiation, and at the end time point. A significant reduction in weight was observed in the DBZ treated animals at the end time point. **B)** H&E and Ki67 staining of sections of intestines from the mice in A, collected at the end time point. Left: representative pictures; right: quantification of Ki positive cells. Five section per mouse were quantified for each treatment group. Inset: few Ki67 positive cells are observed in the crypts of DBZ treated mice, with a lighter staining intensity. A and B: n=5 per group.

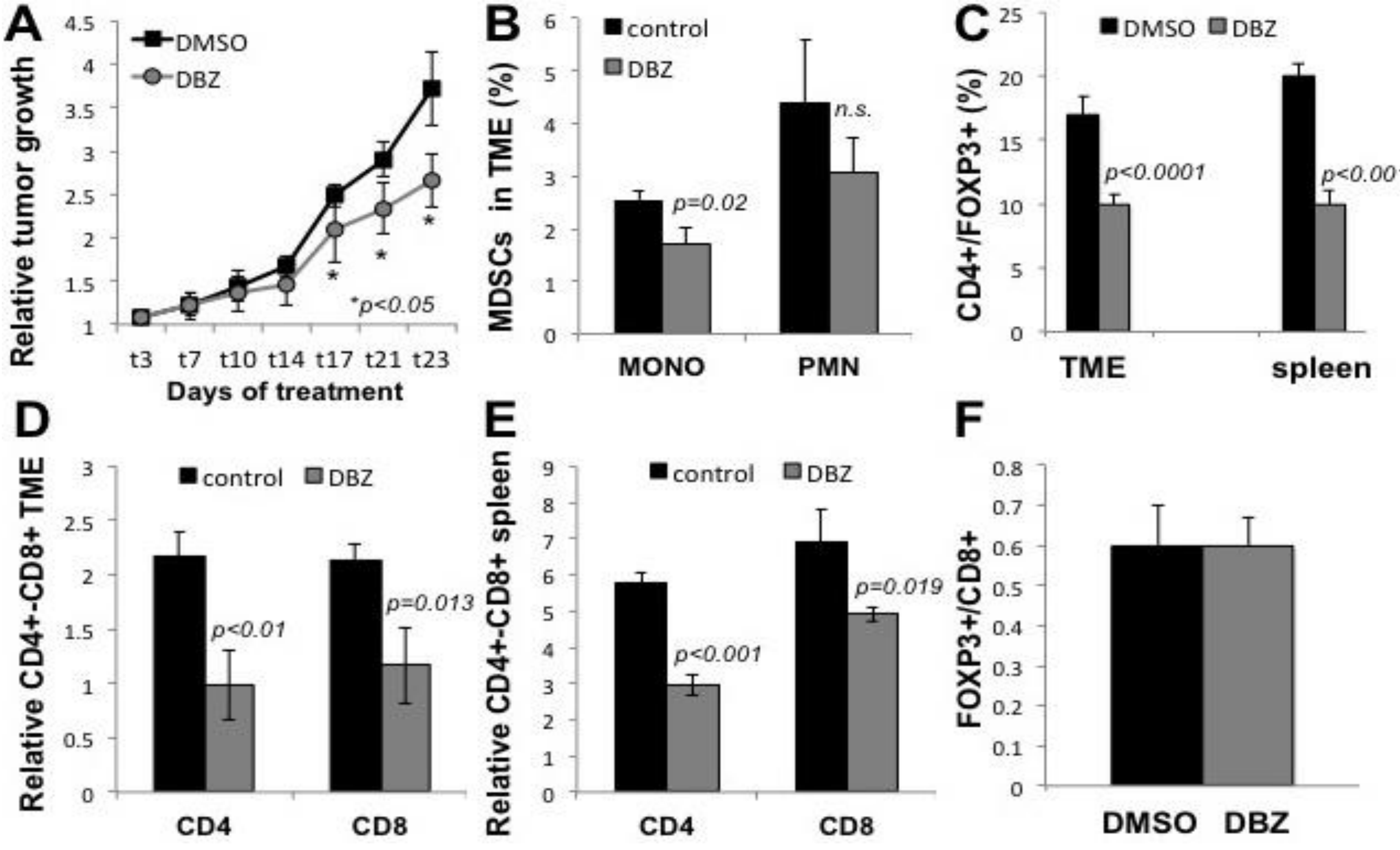

Suppl. Fig. 8: GSI mediated Notch inhibition causes immunosuppression: A) Growth of mouse melanomas induced by a single topical application of 5uM 4-Hydroxitamoxifen on the back of Tyr::CreER; $Braf^{CA/+}$; $Pten^{lox/lox}$ (BRAF/PTEN) transgenic mice. DBZ (10umol/Kg) (dibenzazepine – GSI). Regimen: 3 days on, 4 days holiday. n=10 per group. **B)**% mono- and poly-morphonuclear MDSCs in the tumors in A. **C)** % of Tregs (CD4+/FoxP3+) in the TME and spleen. **D-E)** Relative number of CD4(+) and CD8(+) T cells in the TME and spleen. **F)** Tregs/CD8 ratio in the TME. Data are the mean of two independent experiments.

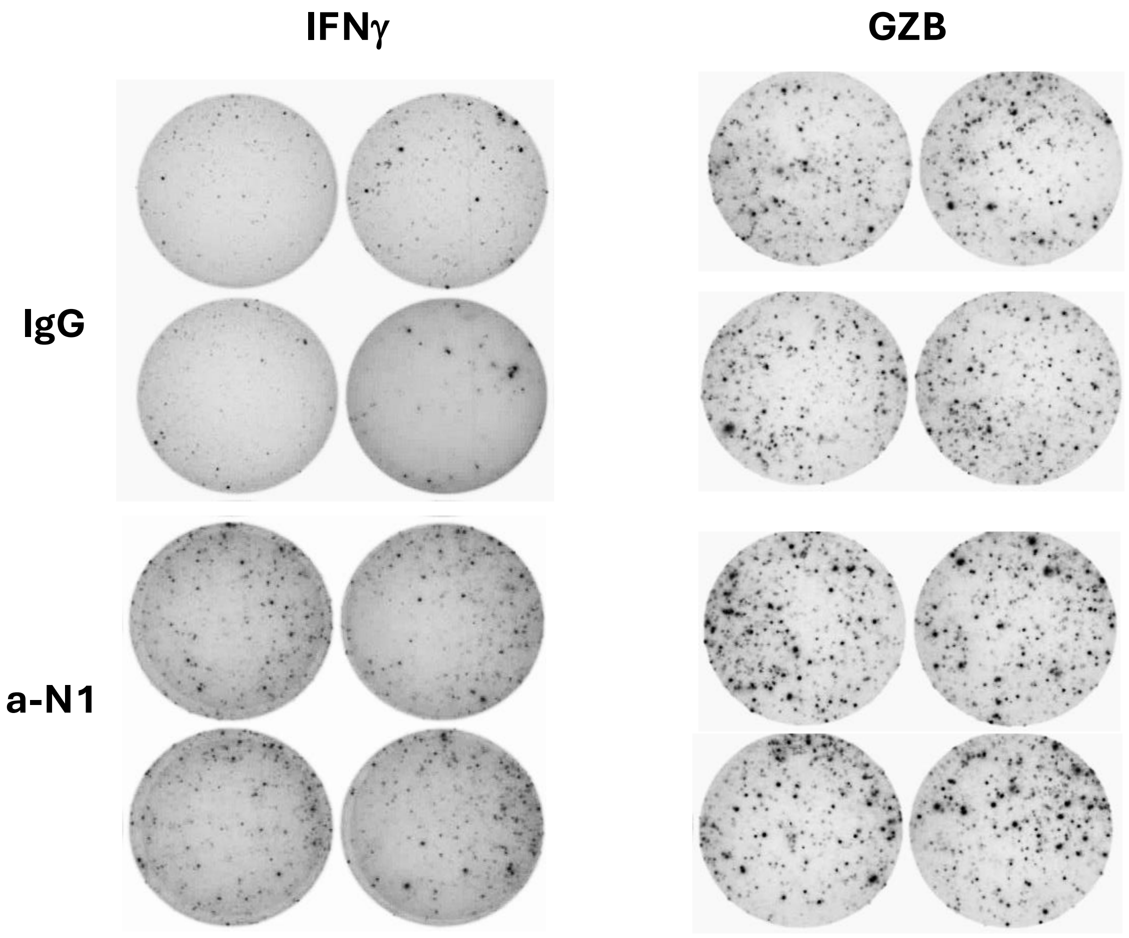

Suppl. Fig. 9) anti-N1 favors IFN© and granzymeB expression in TILs isolated from treated tumors. Tumors were treated with IgG or anti-N1 (10mg/Kg) every other day for for 14 days, TILs were extracted and seeded in vitro, then treated with anti-N1 O/N.

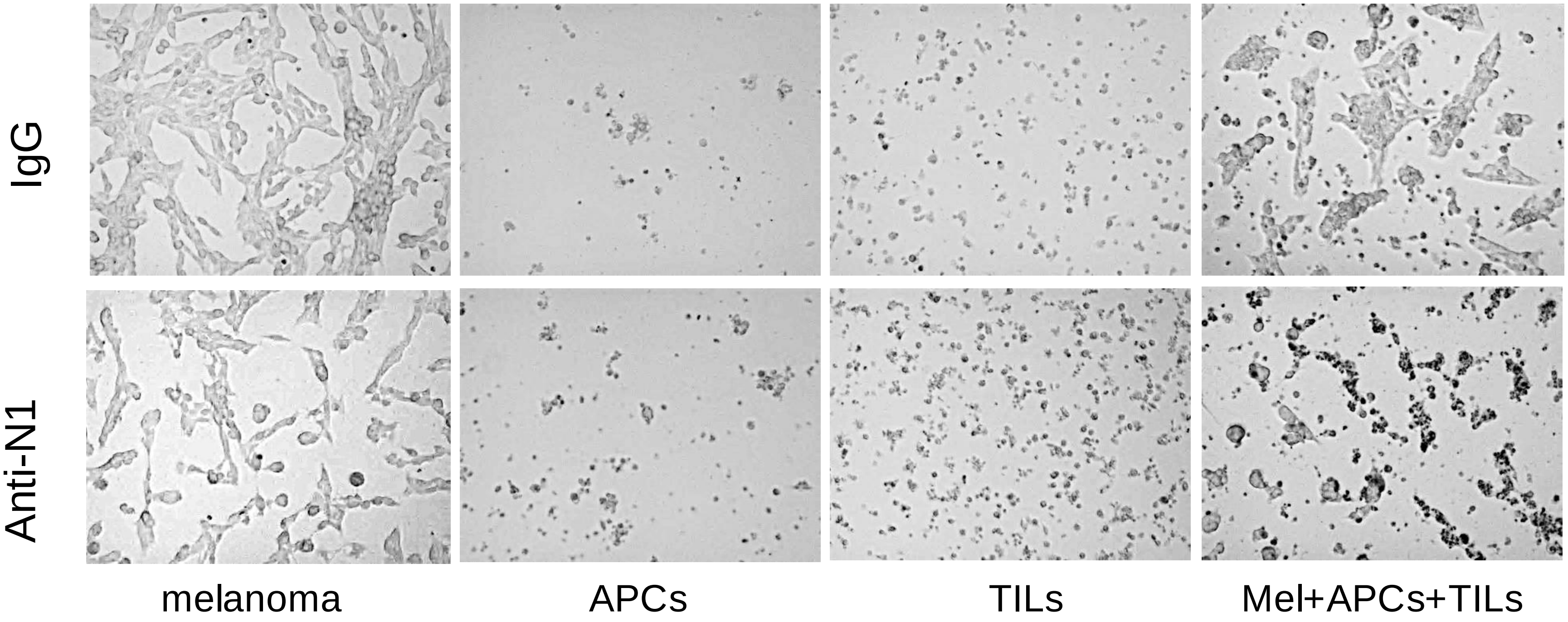

Suppl. Fig. 10) representative brightfield pictures of YUMM2.1 melanoma cells , APCs, TILs and YUMM2.1 melanoma cells in the presence of both APCs and TILs extracted from tumors treated with IgG or a-N1.

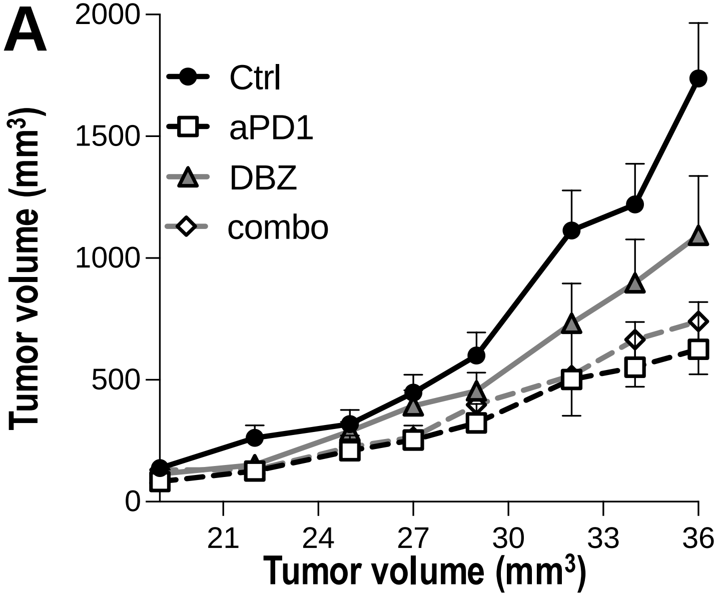

B

T test	**ctrl vs aPD1**	**ctrl vs DBZ**	**ctrl vs combo**	**aPD1 vs combo**	**DBZ vs combo**
19	n.s.	n.s.	n.s.	n.s.	n.s.
22	0.018	n.s.	0.017	n.s.	n.s.
25	n.s.	n.s.	0.042	n.s.	n.s.
27	0.033	n.s.	0.030	n.s.	n.s.
29	0.021	n.s.	0.048	n.s.	n.s.
32	0.020	0.03.	0.014	n.s.	n.s.
34	0.011	0.05.	0.028	n.s.	n.s.
36	0.0016	0.05	0.004	n.s.	n.s.

Suppl. Fig. 11) DBZ does not improve anti-PD1 efficacy. A) tumor growth of YUMM2.1 cells inoculated s.c. into C57 B/L6 mice. Treatment with DMSO/IgG control (10mg/Kg), DBZ (10umol/Kg) or anti PD1 (100ug/mouse) started at day 19 post inoculation, when tumors reached an average volume of 150mm^3. **B)** Student's t test for each time point. n.s.= not significant. n=10 tumors per group.
